# MicroRNA-25/93 induction by Vpu as a mechanism for counteracting MARCH1-restriction on HIV-1 infectivity in macrophages

**DOI:** 10.1128/mbio.01950-23

**Published:** 2023-09-29

**Authors:** Robert Lodge, Zaikun Xu, Mckenna Eklund, Christina Stürzel, Frank Kirchhoff, Michel J. Tremblay, Tom C. Hobman, Éric A. Cohen

**Affiliations:** 1 Laboratory of Human Retrovirology, Institut de recherches cliniques de Montréal (IRCM), Montreal, Quebec, Canada; 2 Department of Cell Biology, University of Alberta, Edmonton, Alberta, Canada; 3 Institute of Molecular Virology, Ulm University Medical Center, Ulm, Germany; 4 Centre de recherche du centre hospitalier universitaire de Québec, Université Laval, Quebec City, Quebec, Canada; 5 Département de microbiologie-infectiologie et immunologie, Faculté de médecine, Université Laval, Quebec City, Quebec, Canada; 6 Department of Medical Microbiology and Immunology, University of Alberta, Edmonton, Alberta, Canada; 7 Li Ka Shing Institute of Virology, University of Alberta, Edmonton, Alberta, Canada; 8 Department of Microbiology, Infectious Diseases and Immunology, Université de Montréal, Montreal, Quebec, Canada; University of Pittsburgh School of Medicine, Pittsburgh, Pennsylvania, USA; University Hospital Heidelberg, Heidelberg, Germany

**Keywords:** human immunodeficiency virus, microRNA, restriction factor, viral glycoproteins, MARCH proteins, HIV-1 countermeasures, Vpu, beta-catenin, macrophages, type-I interferon response

## Abstract

The type 1 interferon-regulated E3 ubiquitin ligase MARCH1 reduces surface expression of HIV-1 envelope glycoproteins (Env) and their packaging into nascent virions, a condition that restricts viral infectivity. However, how HIV-1 counters this restriction, notably during infection of macrophages, remains unclear. Here, we show that the HIV-1 accessory protein Vpu increases the levels of microRNAs-25 and -93 to target *MARCH1* mRNA. By recruiting β-TRCP, a component of the SCF^β-TRCP^ E3 ligase complex that targets phosphorylated β-catenin for degradation, Vpu increases β-catenin levels, which, in concert with TCF4/LEF, drives transcription of the *MARCH1*-targeting microRNAs. This potentiates HIV-1 infectivity as a result of increased Env incorporation into nascent virions. Pharmacological targeting of the β-catenin pathway inhibits Vpu-mediated upregulation of microRNAs-25 and -93 and restores MARCH1 restriction on HIV-1 infectivity. Overall, our findings highlight a novel mechanism by which HIV-1 counteracts MARCH1 by downregulating its expression via Vpu-mediated induction of microRNAs-25 and -93.

**IMPORTANCE:**

In order to efficiently produce infectious viral particles, HIV must counter several restrictions exerted by host cell antiviral proteins. MARCH1 is a member of the MARCH protein family that restricts HIV infection by limiting the incorporation of viral envelope glycoproteins into nascent virions. Here, we identified two regulatory RNAs, microRNAs-25 and -93, induced by the HIV-1 accessory protein Vpu, that downregulate *MARCH1* mRNA. We also show that Vpu induces these cellular microRNAs in macrophages by hijacking the cellular β-catenin pathway. The notion that HIV-1 has evolved a mechanism to counteract MARCH1 restriction on viral infectivity underlines the importance of MARCH1 in the host antiviral response.

## INTRODUCTION

Host organisms have evolved elaborate cell-autonomous defense mechanisms that rely on restriction factors to impede virus replication and spread. These antiviral factors, which frequently target specific components of viruses during their replication cycle, are often induced by interferons (IFNs) as part of the host innate immune response (1). Recently, an emerging class of antiviral restriction factors that target viral envelope glycoproteins and virus entry have been identified (2). These include members of the membrane-associated RING (really interesting new gene)-CH (MARCH) protein family of E3 ubiquitin ligases, which play important roles in multiple biological processes, including turnover of immune regulatory molecules at the plasma membrane (PM) (3). The structurallyrelated human MARCH1, 2, and 8 proteins were initially identified as late-acting restriction factors that impede HIV-1 infectivity by limiting the incorporation of envelope glycoproteins (Env), gp120 and gp41, in nascent virions (4, 5). However, recent evidence suggests that these proteins may have broad-spectrum antiviral activity by restricting incorporation of envelope glycoproteins from other pathogenic RNA viruses, including vesicular stomatitis virus (VSV), lymphocytic choriomeningitis virus, Chikungunya virus, Ebola virus, and severe acute respiratory syndrome coronavirus 2 (SARS-CoV-2) (6 – 9). The antiviral mechanism of MARCH8, which so far has been the most studied member of the MARCH family, appears to involve two distinct modes of restriction depending on the target viral glycoproteins. For example, MARCH8 restricts VSV-G glycoprotein by targeting it for lysosomal degradation following direct ubiquitination of its cytosolic tail (CT)(7, 10). In contrast, restriction of HIV-1 Env appears to be indirect as MARCH8 does not target the CT of this viral glycoprotein. However, both the E3 ubiquitin ligase activity and a CT tyrosine-dependent sorting motif of MARCH8 are required for the intracellular retention and/or degradation of the HIV-1 glycoproteins (7 – 11). Unlike MARCH2 and 8 that are ubiquitous (3), MARCH1 is expressed predominantly in cells of the myeloid lineage, such as monocyte-derived macrophages (MDMs) and monocyte-derived dendritic cells, where it regulates MHC class II expression (12). In contrast to MARCH8, expression of MARCH1 is induced by type I interferon (IFN-I) in MDMs (5). Thus, MARCH1 may serve as an IFN-I-stimulated antiviral factor during HIV-1 infection of macrophages. HIV-1 and other viruses have evolved a complex array of countermeasures to overcome the restriction exerted by IFN-regulated restriction factors (13, 14). However, the mechanism by which HIV-1 counteracts the antiviral activity of the MARCH family of E3 ubiquitin ligases and specifically MARCH1 during infection of macrophages remains unclear.

HIV-1 encodes several accessory proteins, namely Vif, Vpr, Vpu, and Nef, that counter intrinsic host cell defense mechanisms mediated by cellular antiviral restriction factors (15, 16). While neither the Vpr, Vpu nor Nef accessory proteins were found to counter the restriction exerted by MARCH8 on HIV-1 Env and infectivity (4), it is unclear whether these accessory proteins antagonize MARCH1. Notably, HIV-1 Vpu targets the type I IFN-inducible factor BST2 (also called tetherin) to promote efficient virion release (17, 18) and the CD4 receptor to facilitate the expression of conformationally closed Env glycoprotein transport to the cell surface (19 – 21), thus protecting HIV-1-infected cells from antibody-mediated cellular cytotoxicity (22). In order to antagonize these host factors, Vpu exploits the SCF (Skp1/Cullin 1/F-box protein)-β-TrCP (β-transducin repeat-containing protein) E3 ubiquitin ligase complex by recruitment of the β-TrCP subunit to target these proteins for degradation following ubiquitination (23, 24). Other consequences of Vpu binding to β-TRCP are an increase of β-TrCP’s natural cellular targets, including β-catenin, which is regulated by phosphorylation-dependent ubiquitination by SCF^β-TrCP^ (25, 26). Of note, we recently reported that this β-catenin buildup promotes the expression of several microRNAs (miRNAs), including miRNA-93 and miRNA-34c, that target the expression of peroxisome biogenesis factors (27, 28). This led us to hypothesize that changes in miRNA expression during HIV-1 infection of macrophages could alter the production of antiviral restriction factors such as MARCH1.

In this study, we analyzed and compared the mRNA and miRNA transcriptomic profiles of uninfected and HIV-1-infected macrophages. We identified and validated miRNA-25 and miRNA-93 as new negative modulators of MARCH1, which are upregulated early upon HIV-1 infection of macrophages. Inhibiting miRNA-25 and miRNA-93 reduced viral glycoprotein incorporation into virions, a condition that markedly impaired HIV-1 infectivity. Upregulation of these two miRNAs by HIV-1 was dependent on the expression of Vpu. By binding to and displacing the β-TrCP subunit of the SCF^β-TrCP^ E3 ubiquitin ligase complex, we demonstrate that Vpu subverts the host Wnt/β-catenin pathway into enhancing the expression of miRNA-25 and miRNA-93 to counteract the effect of MARCH1 restriction on HIV-1 Env packaging and viral infectivity.

## RESULTS

### miRNA-25 and miRNA-93 are upregulated upon HIV-1 infection of macrophages

**Fig 1:**
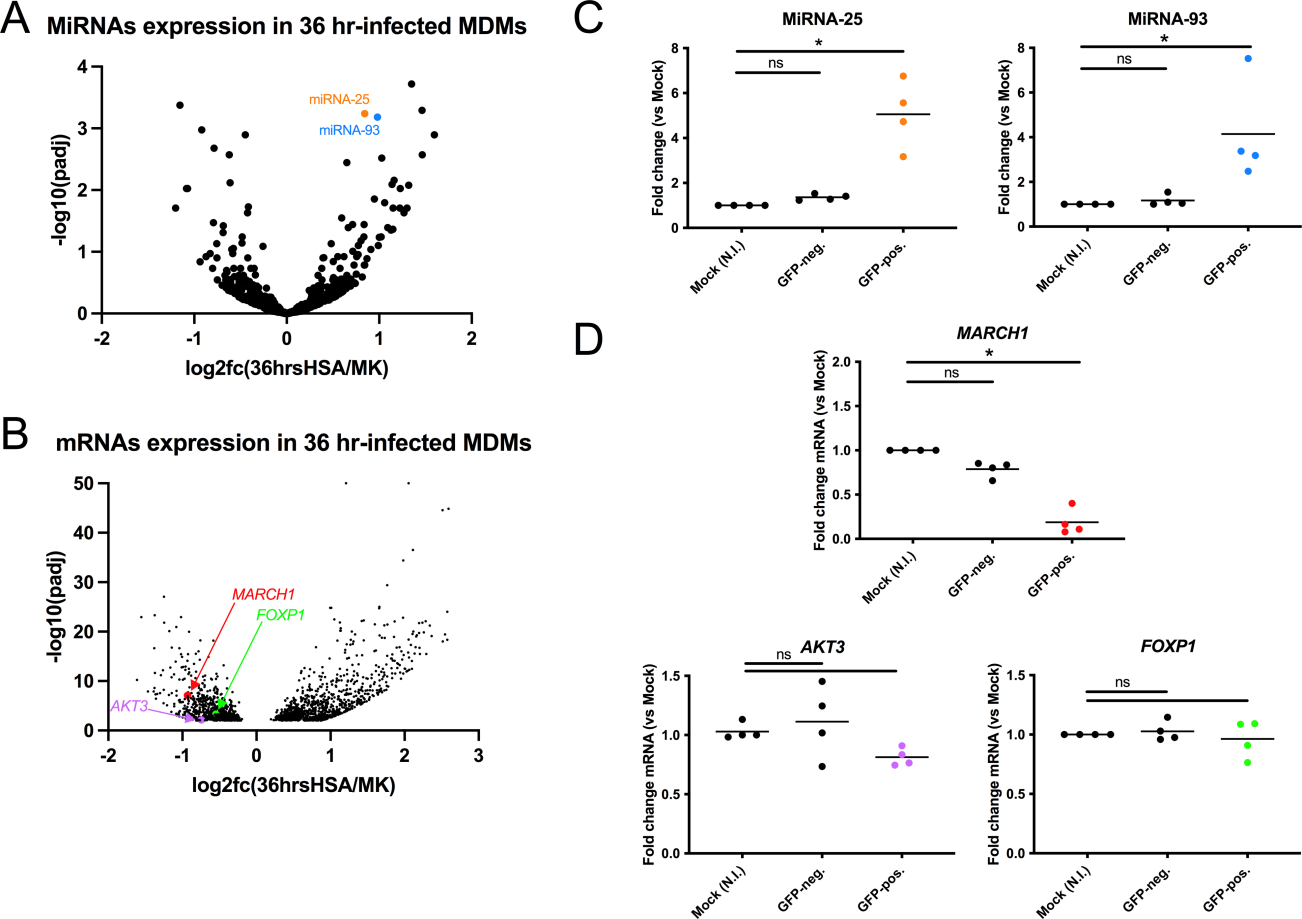
Upregulation of miRNA-25 and miRNA-93 in infected macrophages. See also Fig. S1. Volcano plots representing differential expression (fc= “fold change”; padj= “adjusted *P* value”) of miRNAs (A) or mRNAs (B) in 36 h-productively infected (HSA+) MDMs as compared to mock-infected (MK). MDMs are from four different blood donors. To validate RNAseq/miRNAseq data, miRNA-25 and miRNA-93 (C) and potential target mRNAs expression levels (D) were measured in 36 h-infected MDMs (*n* = 4 different blood donors, as shown) and compared to mock (N.I. = “not infected”). Bars represent means. *P = 0.0286; **P = 0.0079; ns = not significant using the Mann-Whitney test.

miRNAs are single-stranded non-coding regulatory RNA molecules of approximately 22 nucleotides that modulate gene expression in plants, mammals, and even viruses (29). They function by forming duplexes with complementary sequences within a target mRNA, primarily in the 3′ untranslated region (UTR), leading to silencing of the target mRNA by degradation and/or translation block (30). In order to identify miRNAs that are modulated during HIV-1 infection of macrophages, we used previously characterized total RNA samples extracted from MDMs productively infected with a mouse surface heat stable antigen (HSA)-marked HIV-1 (HSA+) for 36 h, or the corresponding bystander (HSA−) MDMs, from four representative donors (31). miRNA expression profiles from these samples, including mock-infected controls, were obtained by next-generation miRNAseq, followed by comparative analyses of miRNA and target mRNA expression. In total, 661 miRNAs were analyzed (Fig. 1A). We focused on the early upregulated miRNAs in the HSA+ macrophages as compared to mock-infected control cells, as these could target possible restriction factors. Out of the 27 miRNAs that were upregulated by at least 1.8-fold (log2fc > 0.84), only 11 were clearly below a DeSeq2 adjusted *P* value of 0.01 (−log10[Padj]) > 2) as shown in Fig. S1. Among these 11 miRNAs, miRNA-25-3p (−log10[*P*_adj_]= 3.24; log2fc = 0.843) and miRNA-93-5p (−log10[*P*_adj_]= 3.18; log2fc = 0.98) stood out as by far as the most abundantly expressed (DeSeq2 base mean > 4,000) (Fig. S1). We thus focused on these two miRNAs that were upregulated in HSA+ macrophages. The RNA transcriptomics data of 1,818 genes (−log10[*P*_adj_] > 2) obtained from total RNAseq analyses (31) of the same samples was also analyzed (Fig. 1B). Importantly, miRNA-25 and miRNA-93 upregulation in HIV-1 productively infected MDMs was validated (Fig. 1C, *P* = 0.0286) by qRT-PCR of RNA samples derived from sorted cells of additional blood donors and using a different reporter HIV-1 construct expressing the green fluorescent protein (GFP).

We then searched online databases (mirDIP, http://ophid.utoronto.ca/mirDIP/) for relevant mRNA targets of miRNA-25 and/or miRNA-93 using the RNA transcriptomics data. Several downregulated mRNAs in HSA+ macrophages, as compared to mock-infected cells, were identified as potential targets of either or both miRNAs: *AKT3* (*P*_adj_ = 0.00710869; log2fc = −0.743112981), *FOXP1* (*P*_adj_ = 0.000483165; log2fc = −0.549687028), and *MARCH1* (*P*_adj_ = 0.000000076; log2fc = −0.931306005) (Fig. 1B). The reduced expression of *MARCH1* in productively infected MDMs was validated (Fig. 1D, *P* = 0.0286) by qRT-PCR. In contrast, we did not detect a significative reduction of *AKT3* or *FOXP1* mRNAs in GFP+ MDMs by qRT-PCR (Fig. 1D). Accordingly, these two genes were thus not further considered in our analyses.

### *MARCH1* is a target of miRNA-25 and miRNA-93

**Fig 2:**
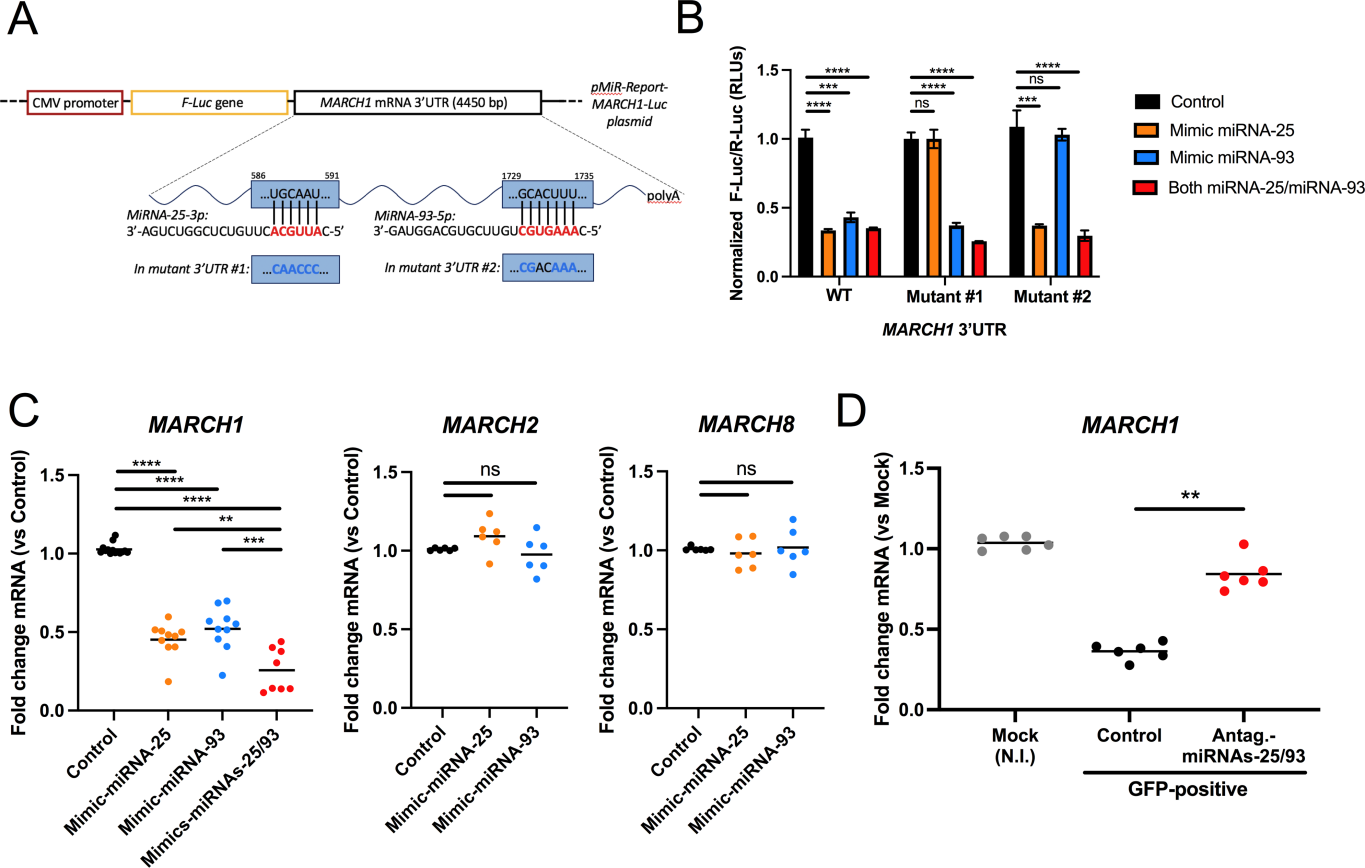
*MARCH1* is a target of miRNA-25 and miRNA-93. (A) The miR-Report system was used to validate the *MARCH1* 3′UTR as a target of miRNA-25 or miRNA-93, respectively. miRNA red nucleotides represent those predicted to interact with the *MARCH1* 3′UTR. WT or mutated (substituted nucleotides in blue) versions of the 3′UTR in the predicted sequences recognized by either miRNA were fused 3′ to the F-*Luciferase* (F-*Luc*) gene. (B) The assay was performed in 293T cells transfected with corresponding mimics, the reporter plasmids, and a Renilla-*Luciferase* (R-*Luc*) normalizing control. Cells were lysed and relative light units (RLUs) were measured and F-Luc normalized to control mimics (*n* = 3). Shown are means ± SD. One-way ANOVA was used for statistical analyses (***P = 0.0004, ****P < 0.0001, ns = not significant). (C) *MARCH1*, *2*, and *8* mRNA levels were measured in IL-10-treated (40 ng/mL) MDMs (*n* = 6–10 blood donors) transfected with the indicated mimics using qRT-PCR. Bars represent means. **P = 0.0014, ***P = 0.0009, ****P < 0.0001, ns = not significant using the Mann-Whitney test. (D) *MARCH1* mRNA levels were measured (qRT-PCR) in sorted, 36 h productively infected (GFP+) MDMs transfected with control or a mix of antagomirs of miRNA-25 and miRNA-93. MDMs were from six different blood donors. Bars represent means. **P = 0.0022 using the Mann-Whitney test.

A search of an online database (TargetScanHuman: www.targetscan.org) suggested that the 3′UTR of *MARCH1* mRNA is a target for both miRNA-25 and miRNA-93. To verify this, we transiently expressed a construct encoding the Firefly *Luciferase* (*F-Luc*) gene fused to the 3′UTR of *MARCH1* (Fig. 2A) in HEK293T cells co-transfected with control miRNA mimics or mimics for either miRNA-25, miRNA-93 or both, and assessed F-Luc activity in cell lysates after 48 h. Compared to controls, F-Luc activity was significantly reduced in cells transfected with miRNA-25 (*P* < 0.0001) or miRNA-93 (*P* = 0.0004) mimics, or combination of both (Fig. 2B). Furthermore, mutations in the *MARCH1* 3′UTR sequences predicted to be targeted by miRNA-25 or miRNA-93 (Fig. 2A), prevented the silencing effect on F-Luc activity (Fig. 2B). Next, MDMs were transfected with miRNA-25 or miRNA-93 mimics (Fig. 2C) and the levels of *MARCH1* mRNA were determined by qRT-PCR. The levels of *MARCH1* mRNA were decreased by ~50% in miRNA-25 (*P* < 0.0001) or miRNA-93 (*P* < 0.0001) mimic-transfected macrophages, compared to controls; a combination of both mimics (*P* < 0.0001) achieved ~75% reduction. In contrast, levels of *MARCH2* and *MARCH8* mRNAs were not affected by these miRNA mimics (Fig. 2C). To assess the effects of miRNA-25 and miRNA-93 during infection, we treated MDMs with a combination of miRNA-25 and miRNA-93 inhibitors (antagomirs) and infected them with a HIV-1 encoding GFP for 36 h. Productively infected (GFP+) cells were then sorted and the levels of *MARCH1* mRNA were assessed by qRT-PCR. Antagomir-treatment strongly diminished the reduction of *MARCH1* mRNA levels in HIV-1-infected cells (Fig. 2D). Altogether, these data indicate that miRNA-25 and miRNA-93 reduce the level of *MARCH1* mRNA in HIV-1 productively infected macrophages.

**Fig 3:**
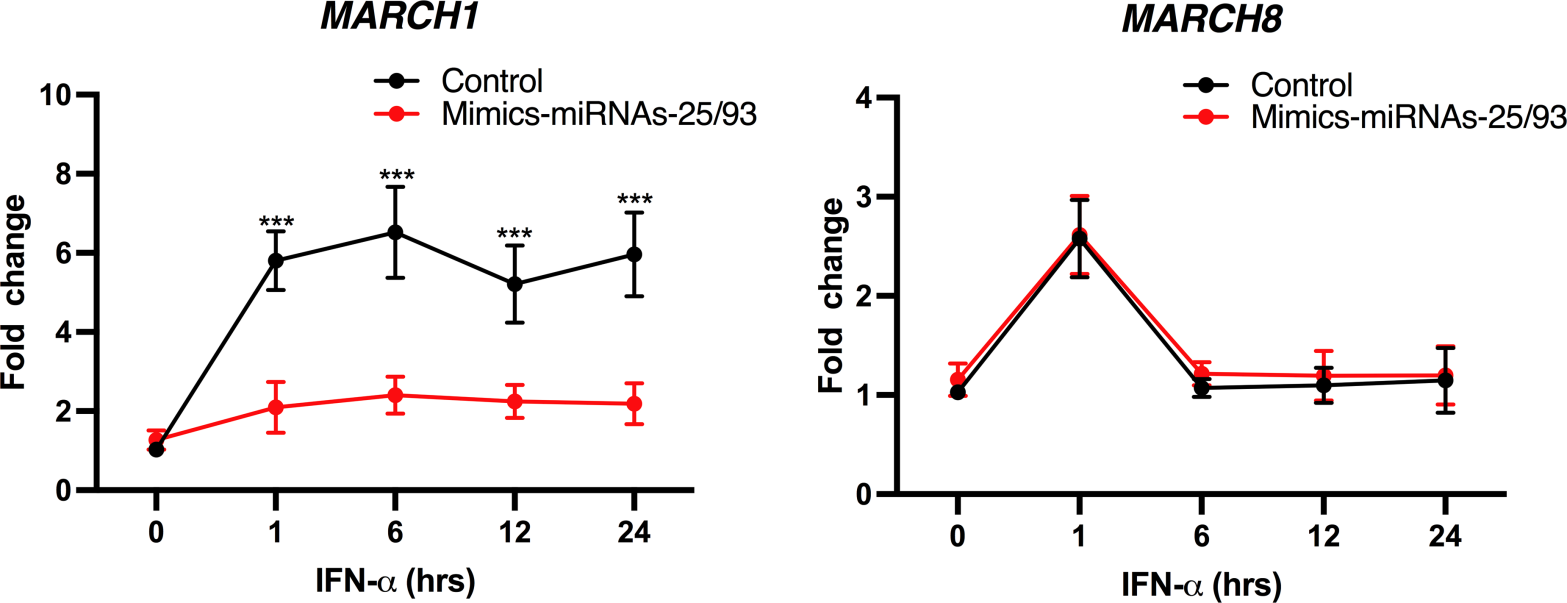
miRNA-25 and miRNA-93 reduce IFN-α-induced *MARCH1* mRNA expression. *MARCH1* or *MARCH8* mRNAs were measured (qRT-PCR) at several time points in MDMs previously treated with IFN-α (100 U/mL) and either control or a mix of miRNA-25 and miRNA-93 mimics. Results are showed for MDMs from six different blood donors. Shown are means ± SD. ***P = 0.000547 using multiple Mann-Whitney tests.

Many genes with antiviral functions are IFN inducible (32). Although HIV-1 has evolved mechanisms to limit the production of IFN-I and expression of IFN-stimulated genes (ISGs) in infected macrophages (14), induction of IFN-I has been reported in these cells (33, 34). Of relevance to this study, *MARCH1* is induced by IFN-I in MDMs (5, 8). Given that our data suggested that *MARCH1* expression in HIV-1-infected MDMs is modulated by miRNA-25 and miRNA-93, we tested whether mimics of these miRNAs could impact the upregulation of *MARCH1* mRNA expression levels in IFN-I-treated MDMs (Fig. 3). Treatment of MDMs transfected with a combination of miRNA-25 and miRNA-93 mimics with IFN-α resulted in a marked (*P* = 0.000547) reduction in *MARCH1* mRNA levels compared to control-treated cells. It is important to note that all our attempts to detect endogenous human MARCH1 proteins in macrophages using a validated anti-MARCH1 antibody (8) in Western blotting were unsuccessful even in IFN-α-treated MDMs. This commercially available anti-MARCH1 antibody (ThermoFisher polyclonal antibody PA5-69223) displayed specific immunoreactivity only when the protein was overexpressed. The lack of antibodies that can specifically recognize endogenous human MARCH1, which undergoes rapid turnover (12, 35, 36), remains a challenge in the field, as stated by Lei et al. (36), and represents a limitation of our study. Although *MARCH8* mRNA levels were slightly induced following 1 h of IFN-I, this induction was not sustained in MDMs, as previously reported (5, 8), and was not affected by the miRNA-25/miRNA-93 mimics. These results support the notion that miRNA-25 and miRNA-93 upregulation during HIV-1 infection is a mechanism to limit the antiviral effects of MARCH1 in macrophages.

### miRNA-25 and miRNA-93 enhance HIV-1 infectivity by promoting incorporation of viral glycoproteins into nascent virions

**Fig 4:**
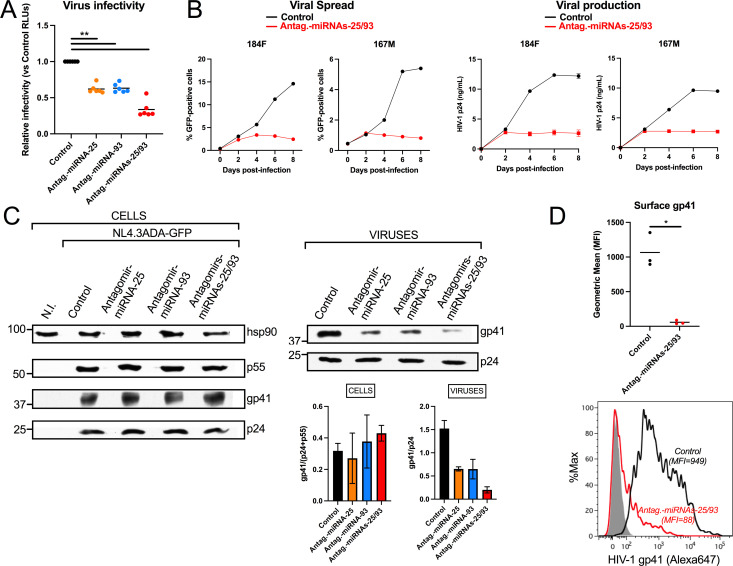
miRNA-25 and miRNA-93 inhibitors reduce HIV-1 infectivity by decreasing Env glycoproteins incorporation into viral particles. See also Fig. S2. (A). Equal p24 amounts (1 ng) of concentrated virus, produced from 36 h-HIV-1-infected MDMs (*n* = 6 blood donors) that had been previously transfected with the indicated antagomirs, were used to infect the TZMbl reporter cell line. The F-Luc activity (RLUs) was assessed in the cell lysates and normalized to that of TZMbl infected with virus from control-treated MDMs. Bars represent means. **P = 0.0022 using the Mann-Whitney test. (B) MDMs of two blood donors were treated with the indicated antagomirs or control and infected with HIV-1 encoding GFP. Spread of infection and viral production over time was determined by evaluating the percentage of GFP-positive cells or the levels of virion-associated p24 in the supernatant at different time intervals. (C) Representative (*n* = 3) Western blots from lysates (left panel) of the MDMs used in “A” and that of their produced virus particles (right panel); quantitative analyses of cellular gp41 levels relative to Gag products as well as the extent of gp41 incorporated into viral particles relative to p24 (*n* = 3) are shown below; means ± SEM. (D) The levels (mean fluorescent intensity [MFI]) of cell surface gp41 were measured in productively (GFP+) HIV-1-infected MDMs treated with controls or the indicated antagomirs by flow cytometry. Bars represent means from MDMs of three blood donors. Representative data from macrophages obtained from one blood donor are also shown below. *P = 0.05 using the Mann-Whitney test.

Using overexpression systems, it has been shown that MARCH1, MARCH2, and MARCH8 downregulate HIV-1 glycoproteins at the cell surface by intracellular retention or targeting of the proteins for degradation, thereby affecting the levels of Env glycoproteins that can be incorporated into nascent viral particles (4 – 10). Here, we used antagomirs of miRNA-25 and miRNA-93, which attenuate the HIV-1-induced reduction of endogenous *MARCH1* (Fig. 2D), to assess the impact of endogenous miRNA-25 and miRNA-93 on the production of infectious particles in macrophages. The initial infection (percentage of GFP+ cells at 36 h post-infection) of MDMs was not affected by prior transfection of control, miRNA-25, miRNA-93, or a mixture of miRNA-25 and miRNA-93 antagomirs (Fig. S2A), nor were levels of virus that were produced (Fig. S2B). Equal amounts of virus produced in each condition (1 ng) were then used to infect reporter cells to assess their level of infectivity (Fig. 4A). We observed a significant (*P* = 0.0022) reduction in infectivity of viruses produced in antagomir-treated MDMs. Specifically, HIV-1 produced in MDMs treated with both miRNA-25 and miRNA-93 antagomirs was ~75% less infectious than virus from control cells (Fig. 4A; Fig. S2C). Macrophages that were treated with the miRNA-25/miRNA-93 antagomirs mix prior to HIV-1 infection displayed an important impairment of viral spread and virus production when compared to control-treated MDMs, suggesting that the benefit of miRNA-25/miRNA-93 upregulation on the spreading of infection is substantial (Fig. 4B). Although the levels of cell-associated Env glycoproteins (as measured by total cellular gp41) were similar in the antagomir and control-treated infected MDMs, incorporation of Env glycoproteins in virions from miRNA-25 or miRNA-93 antagomir-treated macrophages was reduced ~2.5-fold (Fig. 4C). This reduction was even more pronounced in viruses originating from MDMs transfected with the mixture of antagomirs (~8-fold reduction) (Fig. 4C, “viruses” panel and quantification).

To further investigate this effect, we compared the surface expression of gp41 in control or miRNA-25/miRNA-93 antagomir-transfected macrophages infected with GFP-expressing HIV-1 (Fig. 4D; Fig. S2D). The transmembrane gp41 glycoprotein was selected for these analyses to avoid confounding effects resulting from gp120 shedding during infection and cell collection. We observed that antagomir-transfected HIV-1-infected cells express significantly less gp41 on their cell surface as compared to control-transfected cells. Furthermore, confocal imaging showed that, as opposed to the broad staining throughout the cell found in control-transfected infected MDMs, gp41 localized to the perinuclear region in HIV-1-infected MDMs transfected with the miRNA-25/miRNA-93 antagomir mixture (Fig. S2E and F). The lower levels of Env on the cell surface and decreased incorporation into HIV-1 particles are coherent with the reduced infectivity of virions released from antagomir-treated cells (Fig. 4A) and consistent with the reported effects of MARCH proteins on HIV-1 Env expression and virion incorporation (7, 10). Overall, these results support the notion that upregulation of miRNA-25 and miRNA-93 expression during HIV-1 infection of macrophages enhances virus infectivity by countering the ability of MARCH1 to inhibit the incorporation of Env glycoproteins into virions.

### miRNA-25 and miRNA-93 expression are upregulated by HIV-1 Vpu

**Fig 5:**
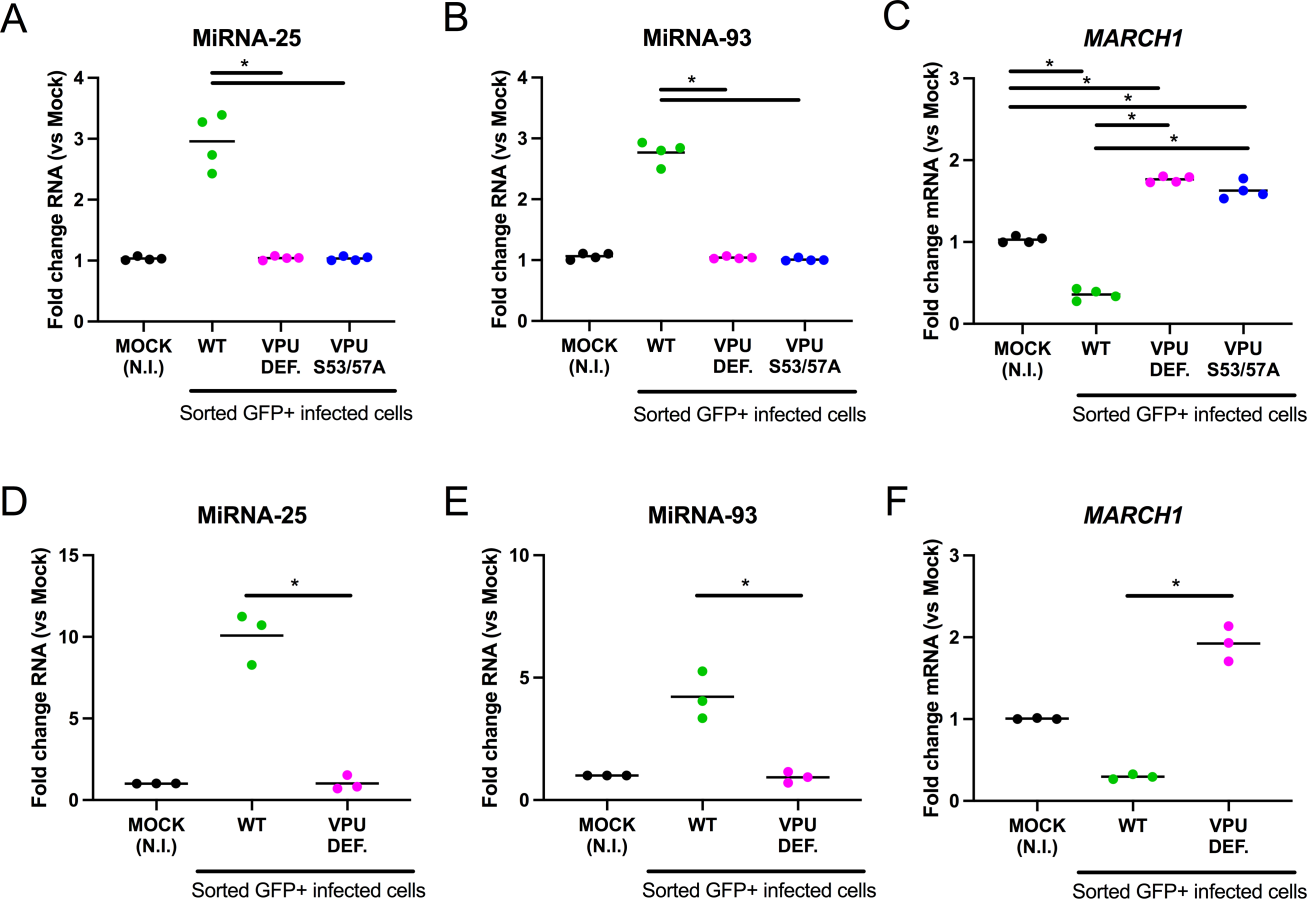
miRNA-25 and miRNA-93 are induced by HIV-1 Vpu. See also Fig. S3. The described RNAs were measured (qRT-PCR) in sorted, 36 h productively infected (GFP+) MDMs, infected with the indicated HIV-1 WT or Vpu-mutant (Vpu def. = Vpu defective; panels A–C, NL4-3-ADA-GFP-IRES-NEF-based; panels D–F, YU2c-IRES-GFP-based) viruses. MDMs are from three or four different blood donors. Bars represent means. *P = 0.0286 using the Mann-Whitney test.

We have previously shown that miRNA-93 is among a group of miRNAs upregulated by the HIV-1 accessory protein Vpu via a mechanism that involves the recruitment of β-TrCP. The interaction is dependent upon a highly conserved motif (DS_52_GNES_56_) in Vpu that is necessary for β-TrCP binding and results in upregulation of the Wnt/β-catenin signaling pathway which drives expression of miRNA-93 (27). We therefore determined whether Vpu also induces miRNA-25 (Fig. 5). MDMs were infected with either wild-type (WT) viruses, Vpu-defective viruses, or viruses encoding a mutant Vpu defective for β-TrCP recruitment (Vpu-S53/57A, based on the Vpu encoded by the CCR5-tropic ADA strain) (23). Infected GFP+ cells were sorted and miRNA-25 expression levels were determined. MDMs productively infected with WT (HIV-1 ADA Env) expressed approximately threefold (*P* = 0.0286) more miRNA-25 than uninfected cells (Fig. 5A). As expected, levels of miRNA-93 were also significantly elevated (*P* = 0.0286) (Fig. 5B). However, neither miRNA-25 or miRNA-93 miRNAs were upregulated in cells infected with either Vpu-deficient viruses or those encoding the Vpu mutant defective for β-TrCP recruitment (Fig. 5A and B). This indicates that like miRNA-93, miRNA-25 expression is modulated by Vpu and its ability to recruit β-TrCP. These results were even more striking using WT and Vpu-defective variants of the R5-tropic HIV-1 neurotropic isolate YU2c, extending these observations to an additional physiologically relevant HIV-1 strain (Fig. 5D and E). Consistent with these observations, downmodulation of the level of *MARCH1* mRNA (*P* = 0.0286) was dependent on the presence of Vpu that had the ability to recruit β-TrCP (Fig. 5C and F). Importantly, we reproducibly detected about twofold higher levels of *MARCH1* mRNA in Vpu-defective or Vpu-S53/57A HIV-1-infected cells, as compared to mock-infected MDMs (*P* = 0.0286) (Fig. 5C and F). The enhancement of *MARCH1* mRNA was dependent on IFN-I release following HIV-1 infection since addition of recombinant vaccinia-encoded IFN-I inhibitor B18R alleviated *MARCH1* upregulation (*P* = 0.05) (Fig. S3A and B).

Unexpectedly, we also found that HIV-2 and simian immunodeficiency virus from macaques (SIVmac) (Fig. S3C through E and F through H, respectively) infections also downregulate *MARCH1* mRNA and upregulate miRNA-25 and miRNA-93, even though these viruses do not encode for a *vpu* gene. These findings highlight the importance of counteracting MARCH1 during primate lentivirus infection of myeloid cells and underline the potential role of miRNA-25/miRNA-93 in this counteraction, although the mechanism driving miRNA-25/miRNA-93 upregulation during HIV-2 or SIVmac infection is likely to be different than in the case of HIV-1.

### miRNA-25/miRNA-93 mediated impact on peroxisome integrity is functionally distinct from their promoting effect on HIV-1 glycoprotein incorporation and infectivity

**Fig 6:**
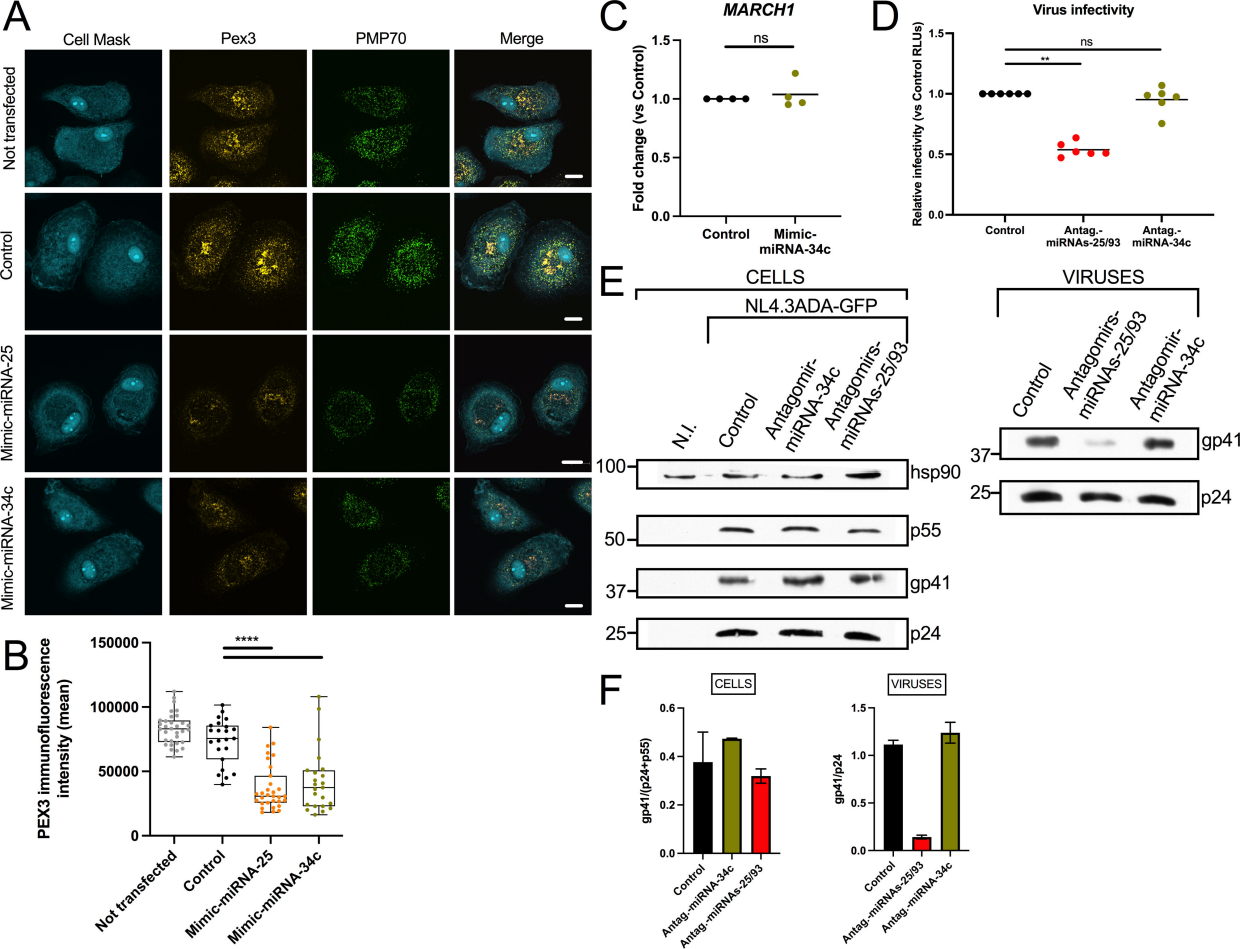
Reduction of peroxisomes by Vpu-regulated miRNAs in MDMs does not impact on Env glycoprotein incorporation into HIV-1 virions and viral infectivity. See also Fig. S4. (A) Representative confocal microscopy images of MDMs transfected with the indicated mimics (48 h) and stained for PMP70 and PEX3, two peroxisomal proteins. Bar = 10 µm. (B) Quantitation of PEX3 intensity in MDMs transfected with the indicated mimics. The box and whiskers (min. to max. points) graph shows the data obtained from *N* = 32 (not transfected), 23 (control), 31 (mimic miRNA-25), or 23 (mimic miRNA-34c) cells; ****P < 0.0001 using Student’s *t* test. (C) IL-10-treated MDMs (from six blood donors) were transfected with either miRNA-34c mimics or controls and the level of *MARCH1* mRNA was measured by qRT-PCR. Bars represent means; *P* = not significant using the Mann-Whitney test. (D) Equal p24 amounts (1 ng) of concentrated virus, produced from 36 h-HIV-1-infected MDMs (*n* = 6 blood donors) that had been previously transfected with the indicated antagomirs, were used to infect the TZMbl reporter cell line. The F-Luc activity (RLUs) was assessed in the cell lysates and normalized to that of TZMbl infected with virus from control-treated MDMs. Bars represent means. **P = 0.0022, ns = not significant using the Mann-Whitney test. (E) Representative (*n* = 3) Western blots from lysates of 36 h-HIV-1-infected MDMs that had been previously transfected with the indicated antagomirs, and that of their produced virus particles. The levels of cellular gp41 and that of gp41 incorporated into viral particles relative to Gag-related proteins are shown in panel F (means ± SEM).

In addition to miRNA-93, we reported that miRNA-34c is among the Vpu-upregulated miRNAs that reduce the level of peroxisomal proteins, thus affecting peroxisome integrity in cells (27). We confirmed the effect of miRNA-34c on peroxisomes by imaging peroxisomal proteins PMP70 and PEX3 in miRNA-34c mimic-treated MDMs (Fig. 6A). We also observed a loss of peroxisomes in miRNA-25 mimic-transfected MDMs (Fig. 6A), suggesting that like miRNA-93 and miRNA-34c, miRNA-25 targets peroxisomal proteins. Quantification of PEX3 immunofluorescence intensity in miRNA-25 mimic-transfected macrophages indicated a loss of peroxisomes as compared to control-treated cells (*P* < 0.0001, Fig. 6B). However, unlike miRNA-25 and miRNA-93, we determined that miRNA-34c does not target *MARCH1* mRNA, as measured by qRT-PCR in miRNA-34c mimic-transfected MDMs (Fig. 6C). We then took advantage of the fact that miRNA-34c targets mRNAs encoding peroxisome biogenesis factors, but not *MARCH1* mRNA, to test whether the effect of miRNA-34c or miRNA-25 on peroxisomes had any consequences for HIV-1 infectivity. Transfection of miRNA-34c antagomirs did not affect HIV-1 particle production in MDMs (Fig. S4A), nor their infectivity (Fig. 6D; Fig. S4B) or the levels of Env glycoproteins incorporated into viral particles (Fig. 6E and F). These observations suggest that disruption of peroxisome integrity has no effect on Env glycoprotein incorporation into virions and HIV-1 infectivity. Therefore, miRNA-25- and miRNA-93-mediated differences in viral glycoprotein incorporation and virus infectivity are primarily due to their targeting of *MARCH1*.

### MARCH1 restriction of HIV-1 infectivity is countered by Vpu-mediated upregulation of miRNA-25 and miRNA-93

**Fig 7:**
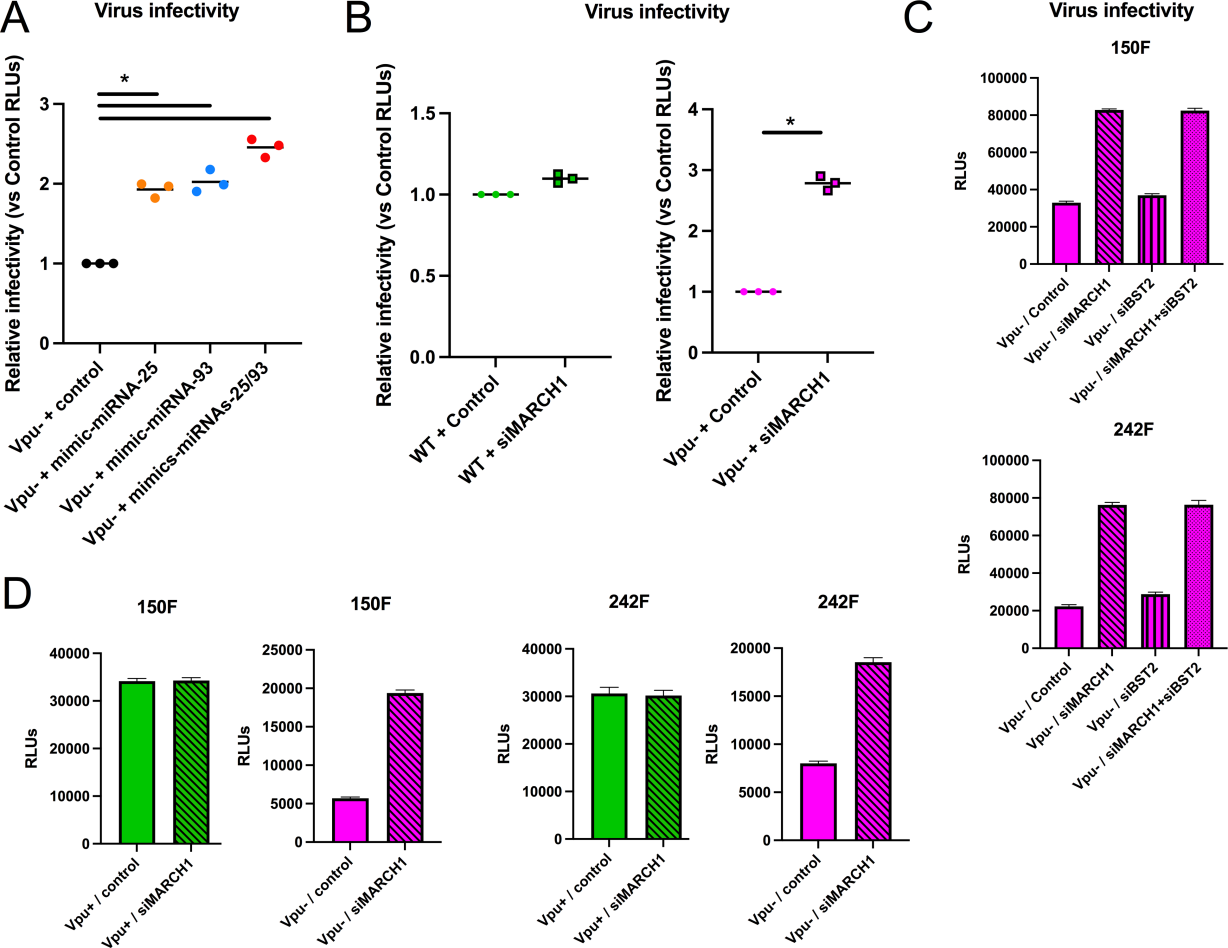
*MARCH1* expression restricts HIV-1 infectivity in macrophages, an effect counteracted by Vpu-mediated upregulation of miRNA-25 and miRNA-93. See also Fig. S5. (A) MDMs were infected with Vpu-defective (Vpu−) HIV-1 viruses and transfected with controls or miRNA-25 or/and miRNA-93 mimics individually or in combination. (B) MDMs were treated with control or siRNAs for *MARCH1* and infected with either WT or Vpu-defective (Vpu−) HIV-1 viruses. (C) MDMs were treated with either control, or siRNAs for either *MARCH1* or *BST2* or a combination of both, and infected with Vpu-defective (Vpu−) HIV-1. Following steps for panel A or B, MDMs were washed, and after 48 h, GFP-positive cells sorted, their total RNAs extracted and supernatants recovered. In panel C, MDMs were washed, and unsorted cells along with supernatants recovered after 48 h. In all cases, released viruses in cleared supernatants were then quantified and normalized (to 1 ng) by ELISA (for HIV-1 capsid protein p24) and virus infectivity was assessed using the TZMbl reporter cell line. (D) MDMs were treated as in panel B, except that following infection (36 h) and washes, TZMbl reporter cells were directly added to the MDMs to determine HIV-1 cell-to-cell transmission; all cells were lysed following an additional 36 h of culture and F-Luc activity measured in lysates. Relative RLUs (vs control) were compiled from MDMs of three (A and B) blood donors; in panels C and D, RLUs are shown for MDMs from two individual blood donors. Bars represent means. *P = 0.05 using the Mann-Whitney test.

Our results suggested that miRNA-25 and miRNA-93 exert their enhancing effect on HIV-1 infectivity by downregulating *MARCH1* expression. To further examine this, we transfected miRNA-25 and/or miRNA-93 mimics into MDMs infected with Vpu-defective viruses, bypassing HIV-1 induction of these miRNAs, and then measured the infectivity of progeny viruses. Transfection of miRNA mimics into MDMs consistently resulted in a twofold increase in the infectivity of Vpu-defective viruses (*P* = 0.05, Fig. 7A; Fig. S5A and B), a context that was associated with the downregulation of the level of *MARCH1* mRNA (*P* = 0.05, Fig. S5C).

To further examine the impact of MARCH1 downregulation by miRNA-25 and miRNA-93 on HIV-1 infectivity, we treated MDMs with control or small interfering RNAs (siRNAs) that target *MARCH1*, prior to infection with WT or Vpu-defective HIV-1 and determined the infectivity of progeny viruses (Fig. 7B; Fig. S5E). *MARCH1* depletion (Fig. S5F) had little effect on the infectivity of WT viruses (Fig. 7B; Fig. 5SE), but resulted in a threefold increase of viral infectivity of Vpu-defective viruses (*P* = 0.05, Fig. 7B; Fig. S5E). It is important to note that infection of macrophages with Vpu-defective virus led to a ~5-fold decrease in virus production due to the restriction exerted by BST2 on virus release (Fig. S5D); to avoid confounding effects resulting from BST2 restriction, viral infectivity was compared separately on WT and Vpu-defective viruses using the virus from untreated siRNA cells as control. We also tested if the increased viral infectivity of Vpu-defective viruses resulting from siRNA-directed *MARCH1* depletion was BST2 dependent. To this end, we knocked down both *MARCH1* and *BST2* (Fig. S5G through I) in Vpu-defective virus-infected MDMs and determined the infectivity of progeny viruses. As shown in Fig. 7C, the effect of siRNA-directed depletion of *MARCH1* on Vpu-defective virus infectivity was found to be basically BST2-independent. Finally, since HIV-1 cell-to-cell transmission is an important mode of viral dissemination (37), we also investigated whether the MARCH1 restriction impacted this type of viral transmission. MDMs were si*MARCH1*-treated, infected with either WT or Vpu-defective viruses, and then cocultured with TZMbl reporter cells for 36 h prior to assessing the luciferase activity in cell lysates as a measure of cell-to-cell transmission. Infectivity and cell-to-cell transmission of Vpu-defective viruses were similarly impacted by *MARCH1* depletion in this experimental model (compare Fig. 7B and D), while infectivity and cell-to-cell transmission of WT virus were not. It is interesting to note that in this system, the absence of BST2 antagonism by Vpu (Vpu−), in the context of siRNA-directed *MARCH1* depletion, did not appear to have a promoting effect on cell-to-cell transmission (Fig. 7D, compare Vpu+ and Vpu− in the context of siRNA-directed *MARCH1* depletion).

Given that MDM infection by Vpu-defective HIV-1 does not lead to an upregulation of miRNA-25 and miRNA-93 (Fig. 5; Fig. S5F), these combined data support the notion that expression of *MARCH1* restricts cell-free HIV-1 infectivity as well as cell-to-cell transmission. They also further suggest that targeting *MARCH1* by these Vpu-induced miRNAs in macrophages is an important factor in controlling the spreading of infection.

### miRNA-25 and miRNA-93-mediated antagonism of MARCH1 restriction of HIV-1 infectivity is inhibited by Wnt/β-catenin inhibitors

**Fig 8:**
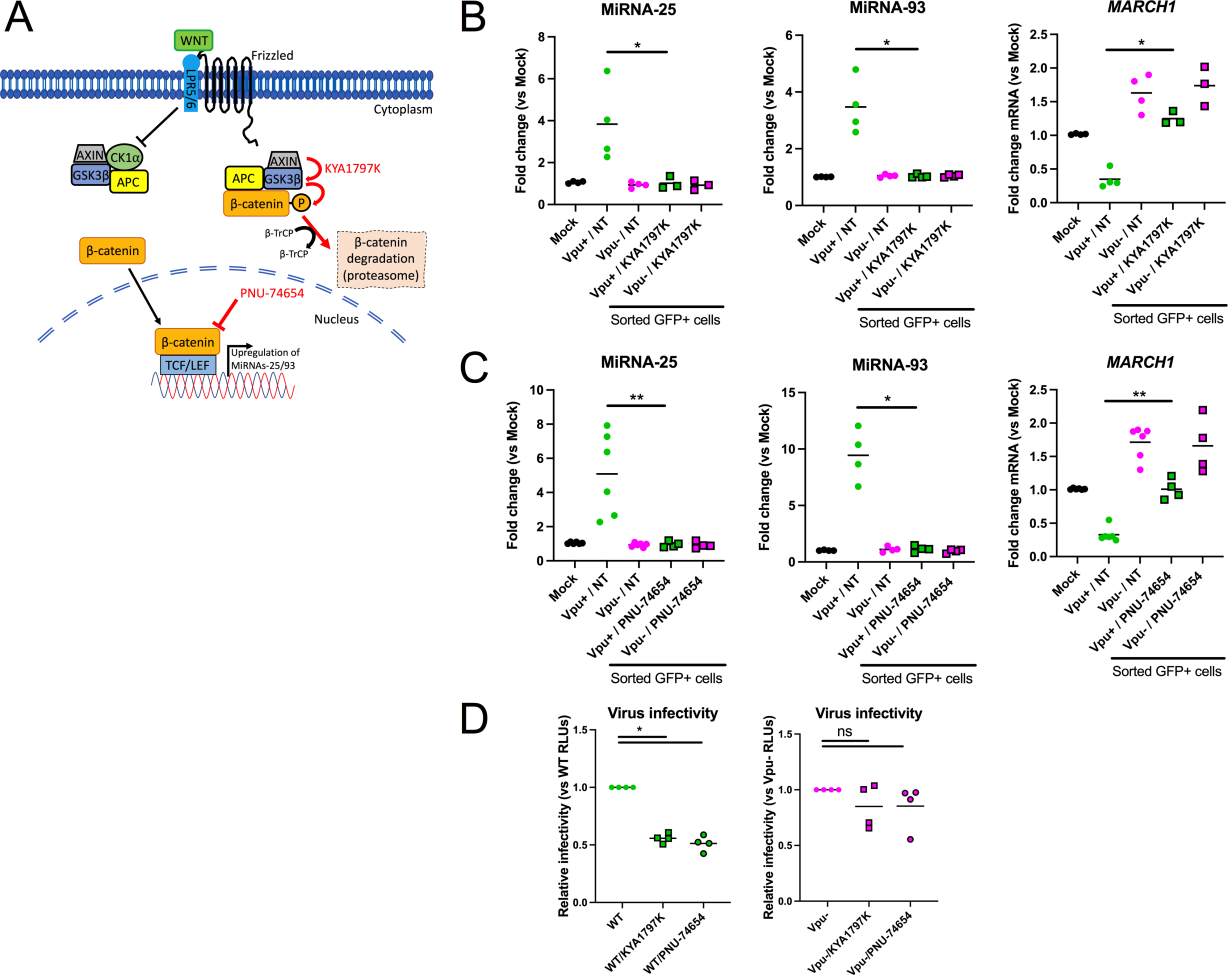
Antagonism of MARCH1 restriction by Vpu-regulated miRNA-25 and miRNA-93 is inhibited by Wnt/β-catenin inhibitors. See also Fig. S6. (A) Graph identifying the steps of the WNT pathway targeted by β-catenin inhibitors KYA1797K and PNU-74654. MDMs (from three to six different blood donors) treated with the β-catenin inhibitors KYA1797K (B) or PNU-74654 (C) were infected with Vpu+ or Vpu-defective (Vpu−) viruses for 36 h, the productively infected (GFP+) MDMs sorted and their total RNA extracted. The levels of miRNA-25, miRNA-93 or *MARCH1* mRNA were then measured by qRT-PCR. Bars represent means. In panel B, *P = 0.0286 (for *MARCH1* and miRNA-25), *P = 0.0143 (for miRNA-93); in panel C, **P = 0.0048, *P = 0.0143 using the Mann-Whitney test. (D) HIV-1 from cleared supernatants were quantified and normalized by p24 ELISA and virus infectivity (1 ng) was assessed using the TZMbl reporter cell line. *P = 0.0143, ns = not significant using the Mann-Whitney test. Relative RLUs (vs vehicle-treated) were compiled from MDMs of four blood donors.

Having previously shown that Vpu-mediated-β-catenin stabilization leads to a TCF/LEF-dependent upregulation of miRNA-93 (27), we next tested if the inhibitory effect of miRNA-25 and miRNA-93 on MARCH1 restriction could be blocked by drugs targeting β-catenin. We first tested how PNU-74654, an inhibitor of β-catenin binding to TCF/LEF (38), or KYA1797K, an enhancer of β-catenin degradation (39), affected the levels of miRNA-25, miRNA-93, and *MARCH1* in MDMs infected with WT or Vpu-defective HIV-1 (Fig. 8A). Neither compound affected the levels of miRNAs or *MARCH1* expression in Vpu-defective virus-infected cells as compared to the mock-infected control (Fig. 8B and C). However, in WT virus-infected cells, both PNU-74654 and KYA1797K significantly reduced miRNA-25 (*P* = 0.0286, KYA1797K; *P* = 0.0048, PNU-74654) and miRNA-93 (*P* = 0.0143, KYA1797K; *P* = 0.0143, PNU-74654) levels, resulting in a concomitant increase in *MARCH1* mRNA (*P* = 0.0286, KYA1797K; *P* = 0.0048, PNU-74654) as compared to vehicle-treated cells (Fig. 8B and C). Moreover, virus recovered from either KYA1797K- or PNU-74654-treated MDMs infected with WT virus (Fig. S6A) was less infectious than that derived from control-treated macrophages (*P* = 0.0143, KYA1797K; *P* = 0.0143, PNU-74654; Fig. 8D; Fig. S6B). Consistent with the central role of Vpu in counteracting MARCH1 restriction (Fig. 8B and C), neither KYA179K nor PNU-74654 treatment affected the infectivity of Vpu-defective virus (Fig. 8D; Fig. S6A and B). Overall, these results indicate that the antagonism of MARCH1 by miRNA-25 and miRNA-93 can be inhibited by pharmacological agents targeting β-catenin, supporting the notion that miRNA-25 and miRNA-93 upregulation is a consequence of the increase in β-catenin brought on by Vpu in HIV-1-infected macrophages and its subsequent association with DNA-binding proteins from the TCF/LEF family.

## DISCUSSION

A growing number of miRNAs have been identified as regulators of HIV-1 replication (29, 30). Several of these small regulatory RNAs are induced by host cells to inhibit virus infection by either directly targeting viral RNA (40) or host factors necessary for virus replication (30, 41 – 43). Conversely, host miRNAs can be hijacked by HIV-1 to promote its replication and spread (30, 44). Here, we describe a novel mechanism whereby two HIV-1-induced miRNAs, miRNA-25, and miRNA-93, allow the virus to avoid a late-stage restriction whereby the IFN-I-regulated MARCH1 E3 ubiquitin ligase downregulates HIV-1 Env glycoprotein from the infected cell surface and inhibits their incorporation into virions. We further show that this is dependent on the ability of HIV-1 Vpu to sequester the β-TRCP subunit of the SCF^β-TrCP^ complex, a situation that results in stabilization of β-catenin and increased transcription of miRNA-25 and miRNA-93.

By comparing the differential expression of miRNAs and mRNAs in productively infected MDMs and HIV-1-exposed but uninfected cells, we found that miRNA-25 and miRNA-93 were among the most abundant miRNAs that were significantly upregulated by HIV-1 infection (Fig. 1). Both miRNAs are part of the miRNA-106b~25 cluster on chromosome 7q22 within the mini-chromosome maintenance complex component 7 (*MCM7*) gene (45). These miRNAs are associated with multiple human cancers (45) and are enhanced by TGF-β signaling (46).

*MARCH1* was identified among the potential miRNA-25 and miRNA-93 targets that were modulated negatively in productively HIV-1-infected macrophages (Fig. 1) and its 3′UTR contains sequences targeted by these two miRNAs, which can downregulate *MARCH1* mRNA in an additive manner (Fig. 2). *MARCH1* is highly inducible by IFN-I in MDMs and we show that the addition of miRNA-25 and miRNA-93 mimics alleviates its expression in IFN-I-treated MDMs (Fig. 3). The downregulation of *MARCH1* expression by these two miRNAs might therefore represent a mechanism exploited by HIV-1 to efficiently infect macrophages, which are increasingly recognized as an important cellular target of HIV-1 at different stages of disease and a potential contributor to the persistent viral reservoir during antiretroviral therapy (47, 48).

There is increasing evidence that MARCH ubiquitin ligases, notably MARCH1, MARCH2, and MARCH8, have broad antiviral activity against RNA virus envelope glycoproteins (4 – 8, 11). In addition to MARCH8 (4), both the overexpression of MARCH1 and MARCH2 have been reported to restrict nascent HIV-1 particle infectivity (5, 9) by limiting incorporation of Env glycoproteins into virions by mechanisms that are not fully defined (7, 8, 10). Our results provide evidence that miRNA-25 and miRNA-93 facilitate Env glycoprotein incorporation into virions and augment HIV-1 infectivity in MDMs by downregulating *MARCH1* expression. Indeed, we show that inhibition of these miRNAs upregulates *MARCH1* mRNA (Fig. 2D) and re-establishes its antiviral activity, efficiently affecting viral spread (Fig. 4). In this regard, HIV-1 displays very few Env spikes on virions (~7–14 spikes per virion) (49 – 51). While the paucity of spikes on HIV-1 particles has been associated with avoidance of humoral immunity, it represents a limiting factor for viral infectivity. Since a number of restriction factors, such as MARCH E3 ligase proteins, target Env packaging into HIV-1, countermeasures to maintain minimal levels of spikes and optimal viral infectivity are likely essential to ensure efficient virus replication and spread.

Recently, it was reported that MARCH8 redirects HIV-1 glycoproteins to the endo-lysosomal compartment, independently of HIV-1 Env CT (7). A tyrosine-based sorting motif in MARCH8 is necessary for its restriction activity on HIV-1 Env, further suggesting that MARCH8 may redirect HIV-1 Env trafficking (10). These studies, based on overexpression systems, suggest that rather than inducing ubiquitination and degradation of certain viral glycoproteins, such as VSV-G, MARCH proteins could redirect transport of others, such as HIV-1 Env. Our results using miRNA-25/miRNA-93 antagomir-treated macrophages are consistent with such a mechanism. In this context, endogenous *MARCH1* expression is upregulated, as opposed to the overexpression of exogenous MARCH proteins used in other studies. In antagomir-transfected MDMs, we did not observe any changes in the total cellular levels of HIV-1 gp41, yet the levels of gp41 in resulting virions were significantly reduced (Fig. 4). In addition, microscopy analyses revealed a redistribution of gp41 to a perinuclear compartment under these conditions (Fig. S2). In that regard, it is interesting to note that MARCH1, like MARCH8, harbors a tyrosine-based sorting motif that may be critical for redirecting the transport of HIV-1 glycoproteins.

We also uncovered a unique antagonism mechanism where the Vpu accessory protein counters the antiviral activity of MARCH1 by downregulating its mRNA levels via the induction of miRNA-25 and miRNA-93 (Fig. 5 and 7). Tada et al. (4) determined that neither Vpu nor Nef affects the antiviral activity of MARCH8. These findings are consistent with our observation that miRNA-25 and miRNA-93 mimics do not alter *MARCH8* nor *MARCH2* mRNA levels (Fig. 2C). Thus, the evolution of a specific HIV-1 countermeasure for MARCH1 through Vpu-dependent modulation of miRNA-25 and miRNA-93 highlights the likely importance of MARCH1 antiviral activity during HIV-1 infection of macrophages. This importance is further highlighted by the Vpu-independent modulation of *MARCH1* by HIV-2 and SIVmac (Fig. S3), the mechanisms of which remain undetermined and warrant further investigation. It is also noteworthy that *MARCH1* is more inducible by IFN-I in macrophages than *MARCH2* or *MARCH8* (5) (Fig. 3). This was particularly evident in our experiments involving Vpu-defective HIV-1 viruses in which *MARCH1* mRNA levels were reproducibly upregulated by an IFN-I-dependent process (Fig. 5; Fig. S3A and B). In that context, it is well established that BST2/tetherin restriction is fully functional, leading to retention of budding particles (17, 18) and innate sensing of viral components by Toll-like receptors (TLRs) upon endocytic uptake and degradation of restricted virions by BST2 (52). This observation suggests that antagonism of BST2/tetherin by Vpu reduces innate sensing and IFN-I production, and also contributes to limiting *MARCH1* expression and its restriction on HIV-1 Env glycoprotein incorporation into virions.

Vpu has multiple functions related to viral glycoprotein transport and particle release, the best characterized being BST2/tetherin antagonism (17, 18) and CD4 receptor degradation, which prevents the intracellular retention of CD4/Env complexes (53). The modulation of miRNA-25 and miRNA-93 expression by Vpu in order to counteract the MARCH1 restriction is thus another addition to the HIV-1 arsenal against the host antiviral response, resulting in enhanced progeny virus infectivity. Interestingly, miRNA-25 and miRNA-93 counteraction of MARCH1 restriction also results in more efficient cell-to-cell viral transmission, as evidenced in our siRNA-directed *MARCH1* depletion assays (Fig. 7D); we also determined that the gain in virus infectivity in the context of *MARCH1* depletion (which can be observed with Vpu-defective viruses) did not involve BST2/tetherin (Fig. 7C). While the downregulation of CD4 by Vpu was previously reported to directly augment viral infectivity by affecting the incorporation of functional Env glycoprotein subunits into virions, these observations were made in T cells or cellular models expressing high levels of CD4 (54 – 56). We cannot entirely exclude that the increased infectivity that we observe with Vpu-proficient virus as compared to Vpu-defective virus may be due in part to Vpu-mediated CD4 degradation and not solely to upregulation of miRNA-25 and miRNA-93. However, given that macrophages express very low levels of CD4 as compared to T cells (57), we believe that the impact of Vpu-mediated CD4 degradation on viral infectivity is likely to play a minor role in this context as compared to the targeting of MARCH1 by the Vpu-regulated miRNA-25 and miRNA-93. It is worth noting that we did not observe any effect on HIV-1 infectivity or viral glycoprotein incorporation into virions linked to the Vpu-mediated reduction of peroxisomes that we previously showed was dependent on induction of miRNAs, including miRNA-93 (27, 28) (Fig. 6). As peroxisomes are important for antiviral defense (58), the inhibitory effect of Vpu on their biogenesis may indicate that they affect HIV-1 replication or pathogenesis through other mechanisms.

We show that Vpu-mediated enhancement of miRNA-25 and miRNA-93 expression is dependent on sequestration of β-TrCP (Fig. 5), a condition that is known to increase the levels β-TrCP substrates, such as β-catenin (25, 27). We previously reported that increased levels of β-catenin result in upregulation of miRNA-93 through its association with DNA-binding proteins belonging to the TCF/LEF family (27, 28). Among these, TCF4 was determined as an important factor in inducing Vpu-dependent induction of this miRNA, among others (27). Since both miRNA-93 and miRNA-25 are part of the miRNA-106b~25 cluster, it is not surprising that both miRNAs are regulated in the same way. Indeed, we show that Vpu-mediated upregulation of miRNA-25 and miRNA-93 can be inhibited by drugs that enhance the degradation of β-catenin or impair β-catenin binding to TCF/LEF (Fig. 8). In doing so, these drugs prevent the antagonism of MARCH1 by miRNA-25 and miRNA-93, supporting the notion that miRNA-25 and miRNA-93 upregulation is a consequence of the increase in β-catenin brought on by Vpu in HIV-1-infected macrophages.

**Fig 9:**
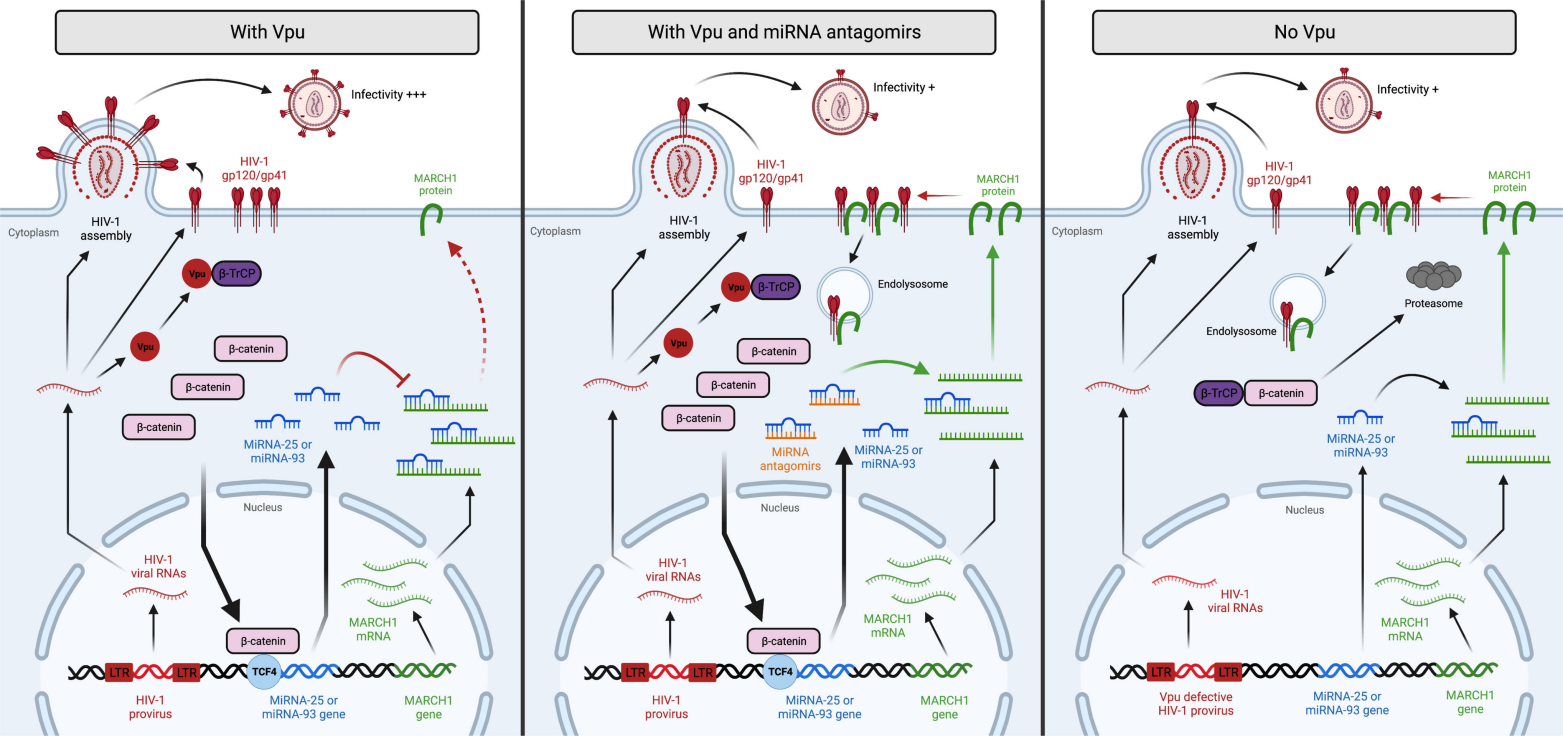
Interplay between Vpu-induced miRNA-25/miRNA-93 and MARCH1 in HIV-1-infected macrophages. Vpu in productively HIV-1-infected macrophages binds and displaces β-TrCP leading to a stabilization of β-catenin. β-catenin translocates to the nucleus where it associates with the DNA-binding protein TCF4 to upregulate miRNA-25 and miRNA-93. The mature forms of these miRNAs then both target *MARCH1* mRNA. Reduced levels of MARCH1 protein leads to less restriction on HIV-1 glycoprotein surface expression and virion packaging, leading to enhanced infectivity of nascent virus particles (left panel). The presence of specific miRNA-25/miRNA-93 antagomirs (central panel) or the absence of Vpu protein (right panel) either inhibits miRNA-25/miRNA-93 effector function (antagomirs) or alleviates miRNA-25 and miRNA-93 expression (Vpu absence), enhancing *MARCH1* expression. In this context, MARCH1 targeting of HIV-1 envelope glycoproteins results in Env intracellular retention and decreased incorporation into nascent virions.

In summary, our results reveal a new mechanism by which HIV-1 Vpu counteracts a host antiviral factor, MARCH1, in macrophages by seizing control of the β-catenin-mediated transcription of miRNA-25 and miRNA-93 (Fig. 9). These findings raise the possibility that HIV-1 accessory proteins, which typically directly target specific host restriction factors (15), may have also evolved other functions to antagonize antiviral factors through indirect means by modifying the expression of miRNAs in the host cell.

## MATERIALS AND METHODS

### Study subjects

Peripheral blood samples were obtained from HIV- and hepatitis C (HCV)-seronegative adults (of either gender).

### Plasmid constructs, MARCH1 3′UTR validation assay, and miRNA mimics and antagomirs

NL4-3-ADA--GFP-IRES-NEF, NL4-3-ADA-GFP-IRES-NEF-ΔVpu (59), NL4-3-ADA-GFP-IRES-NEF-VpuS53/57A (60), VSV-G-expressing (61), and SIV3+ (62) constructs were previously described. The YU2c-IRES-GFP construct was generated by inserting an IRES-GFP fragment into the Asc I and AsiS I restriction sites of the Vpu-positive YU2c construct. A similar strategy was used to create HIV-2-ROD-IRES-GFP and SIVmac239-IRES-GFP. The Vpu-deficient version of YU2c-IRES-GFP was generated by overlap PCR using the appropriate primers (see Table 1). For the miR-REPORT system (Applied Biosystems, Ambion), the plasmid pGL4.70Actin1.2(8) (63), encoding *Renilla* luciferase under the control of the actin promoter, was used instead of pMIR-REPORT-β-gal. In order to generate pMIR-REPORT-MARCH1-Luc, *Pfu* PCR was performed on cDNA of IL-10-activated pooled primary peripheral blood monocytes using primers M1utrSacI-F and M1utrMluI-R (see Table 1), and the fragment equivalent to the full MARCH1 3′ UTR was inserted into the SacI and MluI sites of pMIR-REPORT-Luc. Site-directed mutagenesis of the 3′ UTR of MARCH1 was performed by overlap PCR using primer pairs M1UTR25-MutF and M1UTR25-MutR, or M1UTR93-MutF and M1UTR93-MutR and cloned into pMIR-REPORT-MARCH1-Luc using the same strategy in order to generate pMIR-REPORT-MARCH1mut25Luc, pMIR-REPORT-MARCH1mut93Luc or pMIR-REPORT-MARCH1mut25/93Luc. The 3′-UTR MARCH1 target validation assay was performed as previously described in transfected HEK 293T cells (42) using the pMIR-REPORT-MARCH1-Luc-derived plasmids and the Dual-Glo luciferase assay system (Promega) on a GloMax luminometer (Promega). The miRNA-25-3p (#YM00470873), miRNA-93-5p (#YM00471046) or miRNA-34c-3p (#YM00470582) miRCURY locked nucleic acid (LNA) mimics or the miRNA-25-3p (#YI04100613), miRNA-93-5p (#YI04101031), or miRNA-34c-3p (#YI04102421) antagomirs were purchased from Qiagen (Exiqon). Nontargeting control RNAs were obtained from Dharmacon/GE Healthcare (siGENOME nontargeting 2, #D-001210-02-20) or Ambion/Thermo Fisher Scientific (#AM16104). si*BST2* and si*MARCH1* were obtained from ThermoScientific-Dharmacon (On-Target Plus SMART pool, L-011817-00) and Qiagen (GeneSolution siRNAs, 3550599), respectively.

**TABLE 1:** Primers used in this study (all are 5′ to 3′)

	Mimics (Qiagen-Exiqon)
miRNA25-3p	CAUUGCACUUGUCUCGGUCUGA
miRNA93-5p	CAAAGUGCUGUUCGUGCAGGUAG
miRNA34c-3p	AAUCACUAACCACACGGCCAGG
	Antagomirs (Qiagen-Exiqon)
miRNA25-3p	CAGACCGAGACAAGTGCAAT
miRNA93-5p	TACCTGCACGAACAGCACTTT
miRNA34c-3p	GGCCGTGTGGTTAGTGAT
	To generate pMIR-Report-MARCH1-Luc
M1utrSacI-F	ACGTGAGCTCTGGAACCTGTTGGGAGTTTCTTCACCG
M1utrMluI-R	ACGTACGCGTGGAAACAGCTTTGTTTTTTGCTATCACCAAACC
	To generate pMIR-Report-MARCH1-Luc with mutated 3′UTRs
M1UTR25-MutF	TTAAATAGACAACCCATACATTTGAAGACATTGATA
M1UTR25-MutR	TATCAATGTCTTCAAATGTATGGGTTGTCTATTTAA
M1UTR93-MutF	GGTATCACATGGATCGACAAAGTAATTATCAGTG
M1UTR93-MutR	CACTGATAATTACTTTGTCGATCCATGTGATACC
	To generate YU2c-IRES-GFP-Vpu-minus
VPU_YU2_stop_for	CTTATGGAGATACTTGGGCAGGAG
VPU_YU2_stop_rev	GGACAGGCCTGTGTAATGACTGAGGTGTTAC
VPU_YU2_stop_SOE_rev	CTTGTAACTATTACATTACATGTACTACTTACTGC
VPU_YU2_stop_SOE_for	GTAATGTAATAGTTACAAGTATTAGCAATAGTAGC
	For real-time qPCR of mRNAs
hsMARCH1E1-258F1	TCCCAGGAGCCAGTCAAGGTT
hsMARCH1E2-385R1	CAAAGCGCAGTGTCCCAGTG
hsMARCH2-S3	GTCTCCTTCCGCTACCACTG
hsMARCH2-AS3	TGTCTCCTCTGCCACCTTCT
hsMARCH8 ORF298F1	ACAGGAAGCCTCCACTTCG
hsMARCH8 ORF489R1	GACGTGGAATGTCACTGAG
FOXP1-F	CAAAGAACGCCTGCAAGCCATG
FOXP1-R	GGAGTATGAGGTAAGCTCTGTGG
AKT3-F	ATGAGCGATGTTACCATTGT
AKT3-R	CAGTCTGTCTGCTACAGCCTGGATA
GAPDH-F	GCCATCAATGACCCCTTCAT
GAPDH-R	TTGACGGTGCCATGGAATTT
	For real-time qPCR of microRNAs
miR-25-3p-loop	CAAGTGCAAGATATGTGAGACGTACGTTGAGTACGTCAAGTGAAGTTCAGACC
miR-25-3pFWD	CAAGTGCAAGATATGTGAGACGTACGTTG
miR-25-3pREV	GCATTGCACTTGTCTCGGTCTGA
miR-93-5p-loop	GCACTTTGGCTAGCTATGCAGGTACAGTTGGTACCTGACTCTTGTTCTACCTGC
miR-93-5pFWD	GCACTTTGGCTAGCTATGCAGGTAC
miR-93-5pREV	ACAAAGTGCTGTTCGTGCAGGTAG

### Antibodies and chemicals

The following antibodies were used in Western immunoblotting or confocal microscopy imaging analyses: mouse anti-p24 (#31-90-25; ME Biotech services); mouse anti-hsp90 (#ab-13492; Abcam); Chessie 8 mouse anti-gp41 #13049 (64); rabbit anti-PEX3 (#HPA042830; Sigma); and mouse anti-PMP70 (#SAB4200181; Sigma). The T32 mouse anti-gp41 #11391 (65) was used for the detection of HIV-1 gp41 by flow cytometry and confocal microscopy. Alexa647-labeled mouse anti-human BST2 was from BioLegend (clone RS38E). Fluorochromed Alexa secondary antibodies were from Invitrogen. HIV-1 p24 was measured by ELISA (XpressBio #XB-1000). Additionally, the following reagents were used: HIV-1 integrase inhibitor Raltegravir (Santa Cruz Biotechnology #sc-364600; 20 µM); IFN-I inhibitor B18R (R&D Systems #8185-BR-025; 50 ng/mL); β-catenin inhibitors KYA1797K (Sigma #SML1831; 10 µM) and PNU-74654 (R&D Systems #3534/5; 200 µM); cytokines IFN-α (PBL Assay Science #11100-1; 100 U/mL) and IL-10 (Peprotech #200-10; 40 ng/mL).

### MDM isolation, activation and transfection, and HIV-1 production and infection

MDMs were obtained from peripheral blood mononuclear cells and characterized as previously described (42). Transfection of MDMs with either Exiqon microRNA LNA mimics or inhibitors (antagomirs) was performed using Lipofectamine RNAiMax (Invitrogen) as previously described (42). Following a 3-day incubation, cells were harvested for qRT-PCR or flow cytometry sorting. In some cases, a second transfection was performed. Viruses were produced and titers were determined as previously described (42), using the TZM-bl reporter cell line (66). Macrophages were pre-treated with VSV-G-pseudotyped SIV3+ vectors and then infected with HIV-1 at a multiplicity of infection (MOI) of 1 (or 5 for YU2-based viruses).

### Flow cytometry

Cells were collected following a 15 min, 37°C incubation in phosphate-buffered saline (PBS)-EDTA and gentle scraping. Harvested cells were then fixed with 4% paraformaldehyde in PBS for GFP expression analyses or processed for flow immunocytometry. In this case, macrophages were washed in PBS containing 2% fetal bovine serum (FBS) (FACS buffer) and blocked on ice for 1 h in FACS buffer containing 2% goat serum, 2% rabbit serum, and a mix of human decomplemented plasma. Mouse primary antibodies were added directly into the cells in the blocking solution, incubated 1 h on ice and cells were washed two times in FACS buffer. Secondary fluorescent antibodies were then added for 30 min on ice, and cells were washed and fixed with 4% paraformaldehyde in PBS. Finally, cells were resuspended in PBS-EDTA and analyzed on a BD Fortessa cytometer (BD Biosciences) equipped with appropriate lasers. Detailed analyses were obtained using the FlowJo software package. Geometric mean fluorescence intensities were used.

### Confocal microscopy

MDMs grown on coverslips were processed for confocal microscopy following transfection or infection. Cells were washed in PBS and fixed in 3% paraformaldehyde in PBS at room temperature, permeabilized with 0.2% Triton X-100 for 10 min and Fc receptors blocked either with blocking buffer (a mix of human decomplemented plasma or 1 mg/mL human IgGs in PBS) on ice. Samples were then incubated on ice for 1 h with primary antibodies diluted in blocking buffer, washed three times in wash buffer (PBS containing 2% FBS) and incubated with fluorochrome-labeled secondary antibodies for 1 h on ice, washed three times, and mounted on slides. In some cases, Cell Mask Deep Red stain (ThermoFisher) or DAPI was used to visualize cells. For HIV-1 gp41 staining, samples were examined using a Zeiss 710 confocal microscope using a 40×/1.3 oil PlanApo objective and images were acquired using the Zeiss Zen software package. For peroxisome staining, samples were examined using an Olympus 1 × 81 spinning-disc confocal microscope with a 60×/1.42 oil PlanApo objective. PEX3 immunofluorescence intensity was determined as previously described using Volocity 6.2.1 software (27).

### Sodium dodecyl sulfate-polyacrylamide gel electrophoresis and Western immunoblot analyses

Sodium dodecyl sulfate-polyacrylamide gel electrophoresis of macrophage or virus lysates and immunoblotting were performed as previously described (67). Protein quantification was performed by scanning bands using NIH ImageJ.

### RNA extraction, reverse transcription, and real-time qPCR analyses

Total cellular RNAs were extracted using RNeasy RNA extraction columns (Qiagen) according to the manufacturer’s instructions and stored at −80°C. For the isolation of productively infected (GFP-positive) and bystander (GFP-negative) macrophage populations, MDMs infected with NL4-3-ADA-GFP-IRES-NEF-based viruses (MOI of 1) were sorted using an Influx cell sorter (BD Biosciences), and GFP-positive and GFP-negative cells were directly recovered in RLT lysis buffer as previously described (42). In the case of microRNAs, cDNAs were obtained by using two-tailed qRT-PCR (68); briefly, 100–300 ng of total RNAs were reversed transcribed using SuperScript II reverse transcriptase (Invitrogen) with poly(dT) and specific loop primers for the appropriate microRNAs. For qRT-PCR, cDNA and appropriate primers (see Table 1) were added to SYBR green select master mix (Applied Biosystems) in 96-well plates and run on a ViiA96 thermocycler (Thermo Fisher Scientific). GAPDH was used as a loading control, and ΔΔ*C_T_* variations were calculated.

### RNA-Seq of sorted HIV-1-infected macrophage mRNAs and miRNAs and analysis of their expression in productively infected macrophages

Details on how samples were obtained and processed for comparative mRNA expression of productively infected or bystander versus uninfected (mock infected) macrophage populations and the bioinformatics processing of sequences can be found in Deshiere et al. (31). These same samples were used for miRNAseq performed using the Illumina TruSeq Small RNA system (Illumina Technologies) at the IRCM Molecular Biology and Functional Genomics Core Facility. Specific tagging was used to identify RNA from each blood donor. The resulting library was sequenced at the Core Facility using 50 bp paired-end (PE50) sequencing on a HiSeq 2000 sequencer (Illumina Technologies). Sequences were then processed at the IRCM Bioinformatics Core Facility. Adaptor sequences were trimmed with Cutadapt and analyzed RNAs set at 17–35 base pairs. Alignment and quantification of individual miRNAs were performed using miRDeep2 with the miRbase.org (v.21) database. Differential expression of miRNAs was assessed using adjusted *P* values computed using DESeq2. Volcano plots were generated using GraphPad Prism9 (GraphPad Software, LLC). The online search for miRNA and mRNA pairing was performed using mirDIP (http://ophid.utoronto.ca/mirDIP/).

### Statistics

Statistical analyses were performed in GraphPad Prism9 using the nonparametric Mann-Whitney rank test, Student’s *t* test, or one-way ANOVA. Statistical significance is indicated in the figures (*, *P*  <  0.05; **, *P*  <  0.01; ***, *P*  <  0.001; ****, *P*  <  0.0001).

## Data Availability

Online GEO accession numbers to the mRNA-seq and miRNA-seq data reported in this study are GSE180273 (mRNA-seq) and GSE184665 (miRNA-seq).

## SUPPLEMENTAL MATERIAL

Supplemental material mbio.01950-23-s0001.pdf Figures S1 to S6.

ASM does not own the copyrights to Supplemental Material that may be linked to, or accessed through, an article. The authors have granted ASM a non-exclusive, world-wide license to publish the Supplemental Material files. Please contact the corresponding author directly for reuse.
