# Supplementary material for: MicroRNA-25/93 induction by Vpu as a mechanism for counteracting MARCH1-restriction on HIV-1 infectivity in macrophages
Source: mBio. 2023 Sep 29;14(5):e01950-23. doi: 10.1128/mbio.01950-23 (PMC10653795; doi:10.1128/mbio.01950-23)
Supplement: Supplemental material — Figures S1 to S6. [file mbio.01950-23-s0001.pdf]

MiRNA	log2fc(36hrsHSA/MK)	-log10(Padj)	DeSeq2 base mean
**hsa-miRNA-4792**	1.59739995	**2.894391033**	38.7629013062
**hsa-miRNA-145-5p**	1.465080023	**2.570969974**	8.2205600739
**hsa-miRNA-5100**	1.462900043	**3.29203571**	111.1610031128
**hsa-miRNA-4746-5p**	1.350039959	**3.718598422**	25.4706993103
**hsa-miRNA-5096**	1.318179965	**2.077716302**	10.3184995651
hsa-miRNA-5585-3p	1.302109957	1.707325336	4.9048800468
hsa-miRNA-1246	1.266749978	1.63288833	4.4262700081
**hsa-miRNA-1270**	1.227169991	**2.024797589**	28.5960998535
hsa-miRNA-6501-5p	1.222609997	1.707325336	7.3113698959
**hsa-miRNA-20b-5p**	1.160640001	**2.158532913**	20.1558990479
hsa-miRNA-3939	1.150439978	1.707325336	9.5394296646
hsa-miRNA-3180	1.146950007	1.365088642	6.4254999161
hsa-miRNA-3180-3p	1.146950007	1.365088642	6.4254999161
**hsa-miRNA-301a-5p**	1.13973999	**2.092348975**	15.673500061
hsa-miRNA-7974	1.127210021	1.351393153	11.7735996246
hsa-miRNA-877-3p	1.091709971	1.39066282	9.68309021
hsa-miRNA-7977	1.05964005	1.795931569	336.191986084
**hsa-miRNA-760**	1.027609944	**2.519571926**	30.3609008789
hsa-miRNA-937-3p	1.020230055	1.240203778	7.4914197922
hsa-miRNA-6087	1.002089977	1.234313576	13.7462997437
hsa-miRNA-4521	0.990283012	1.102360274	41.6949005127
**hsa-miRNA-93-5p**	0.980381012	**3.180831304**	4133.2099609375
hsa-miRNA-222-5p	0.948122978	1.853884374	1229.2299804688
hsa-miRNA-155-3p	0.910776019	1.037785551	12.5967998505
hsa-miRNA-1273h-5p	0.867715001	0.8870165	27.2656002045
hsa-miRNA-1292-5p	0.843047977	0.786012325	11.0783996582
**hsa-miRNA-25-3p**	0.843029022	**3.239836208**	10923.599609375

Figure S1 (related to figure 1)

Figure S1; related to figure 1: Table of the highest upregulated miRNAs in HSA+ sorted macrophages (as compared to uninfected controls). Shown are the miRNAs upregulated with a log2 fold change (log2(fc)) > 0.84; those with a -log10 of the DeSeq2 adjusted P value (-log10(Padj)) > 2 are highlighted in **bold**. In red, miRNA-25-3p and miRNA-93-5p are the only miRNAs with DeSeq2 base means > 4000.

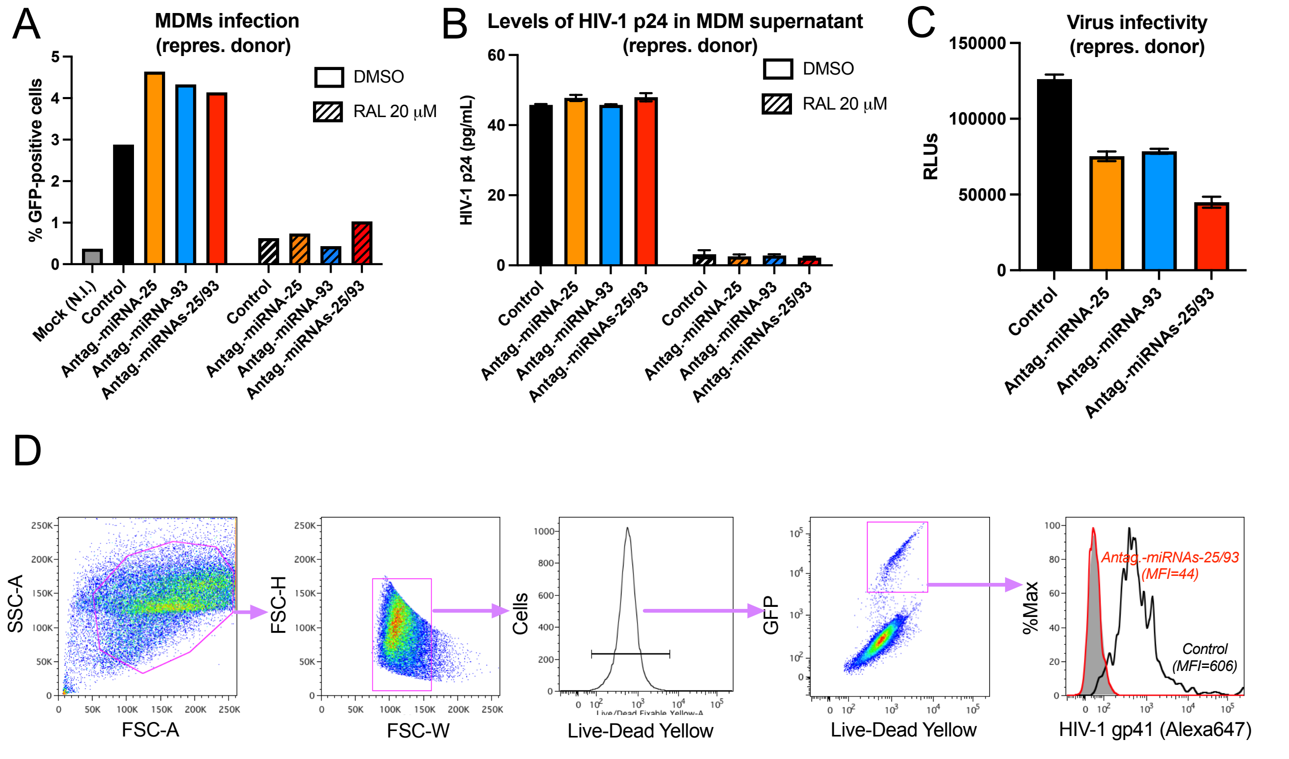

Figure S2 (A-D) (related to figure 4)

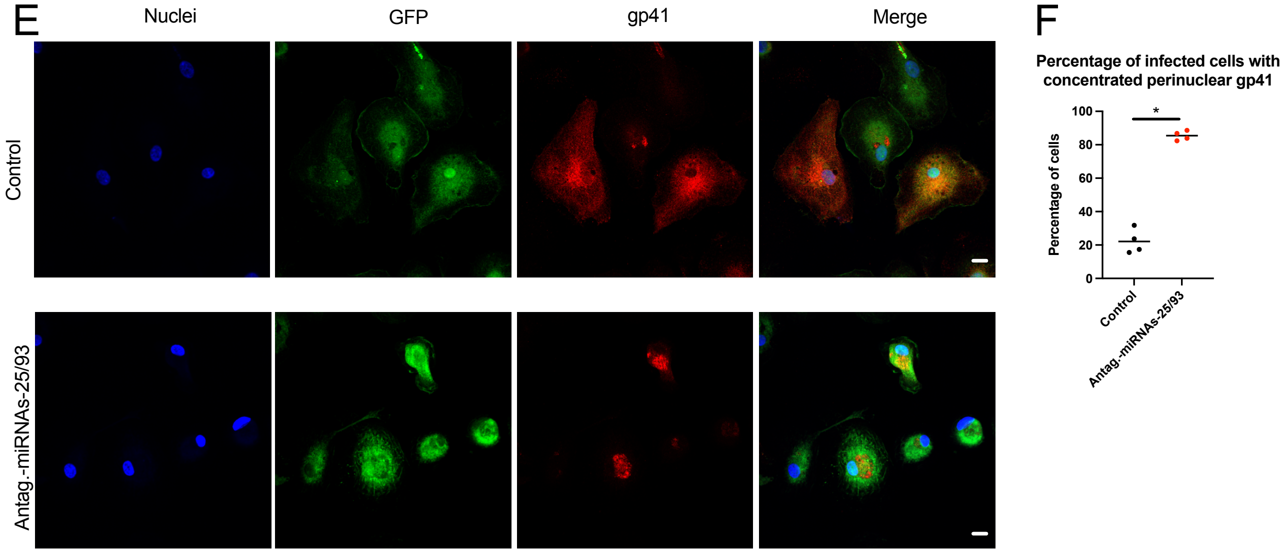

Figure S2 (E-F) (related to figure 4)

Figure S2; related to figure 4: A-C. Representative data obtained from MDMs of one blood donor are shown (related to figure 4A): Macrophages were treated with the indicated control or antagomirs and infected with GFP-expressing HIV-1 virus, washed, and supernatants recovered after 36 hrs of infection. The levels of GFP-expressing MDMs were measured by flow cytometry (A). Viruses in cleared supernatants were concentrated and the levels of HIV-1 p24 determined by ELISA (mean±SD; B). Included were cells treated with the HIV-1 integrase inhibitor Raltegravir (RAL, 20 μM) to control for input virus background. Equal amounts of virus (1 ng) were used to infect the TZMbl reporter cell line. F-Luc activity was measured in cell lysates (mean±SD; C). D. Labeling strategy for flow cytometry analyses of cell surface levels of HIV-1 gp41 (described in figure 4D). E. Representative confocal microscopy images of MDMs transfected with either control or a mix of antagomirs for miRNA-25 and miRNA-93, infected with HIV-1 for 36 hrs, and stained for HIV-1 gp41. Bar = 10 μm. F. Percentage of infected (GFP+) MDMs showing concentrated perinuclear staining for HIV-1 gp41 (MDMs from 4 blood donors). *p= 0.05 using the Mann Whitney test.

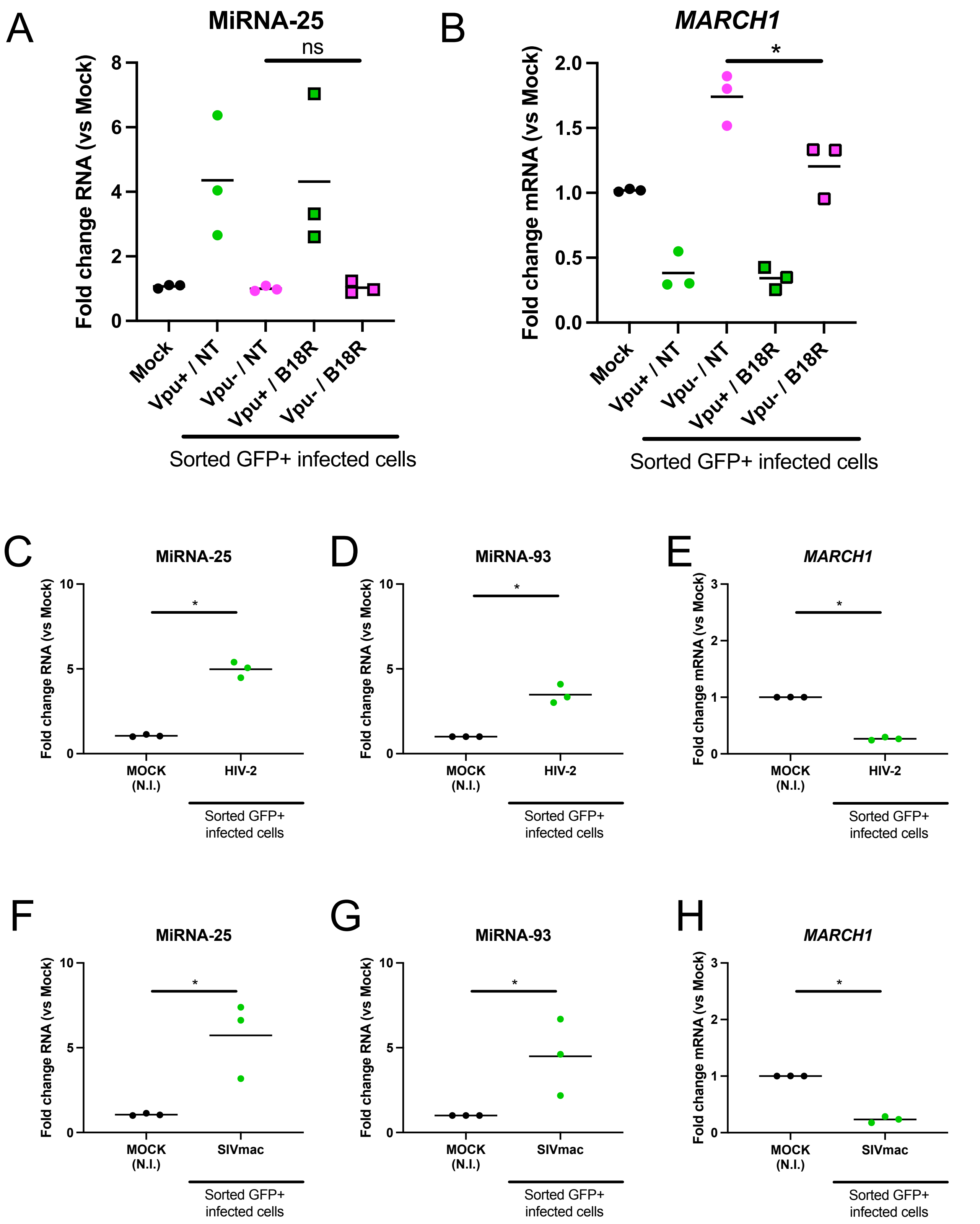

Figure S3 (related to figure 5)

Figure S3; related to figure 5: A and B. Macrophages were treated with vehicle or the type I IFN inhibitor B18R (50 ng/ml) prior and during infection with the indicated viruses. GFP-positive cells were sorted after 36 hrs of HIV-1 infection, their total RNA extracted and the levels of miRNA-25 and *MARCH1* mRNA measured by qRT-PCR. Bars represent means. *p= 0.05 and ns=not significant using the Mann Whitney test. C to H. The described RNAs were measured (qRT-PCR) in MDMs productively infected (GFP+) with VSV-G-pseudotyped naturally Vpu-lacking HIV-2-ROD-IRES-GFP (C-E) or SIVmac239-IRES-GFP (F-H) viruses. MDMs are from 3 different blood donors. Bars represent means. *p= 0.0286 using the Mann Whitney test.

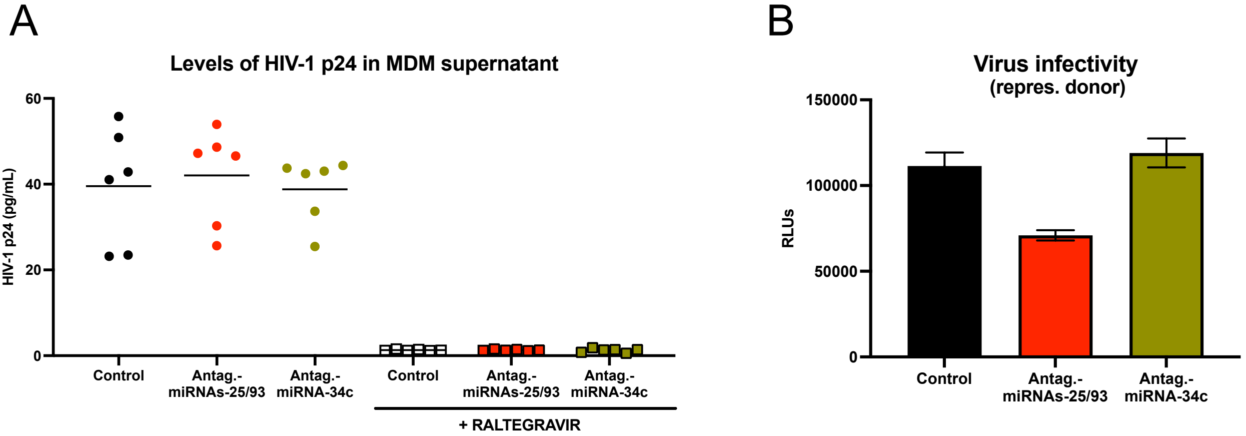

Figure S4 (related to figure 6)

Figure S4; related to figure 6: Macrophages were treated with the indicated control or antagomirs and infected with HIV-1, washed, and supernatants recovered after 36 hrs of infection. Viruses in cleared supernatants were concentrated and the levels of HIV-1 p24 determined by ELISA. (A) Equal amounts of these viruses (1 ng) were used to infect the TZMbl reporter cell line and the F-Luc activity measured in the cell lysates is shown in a representative experiment (B) as well as in figure 6D. Bars represent mean±SD.

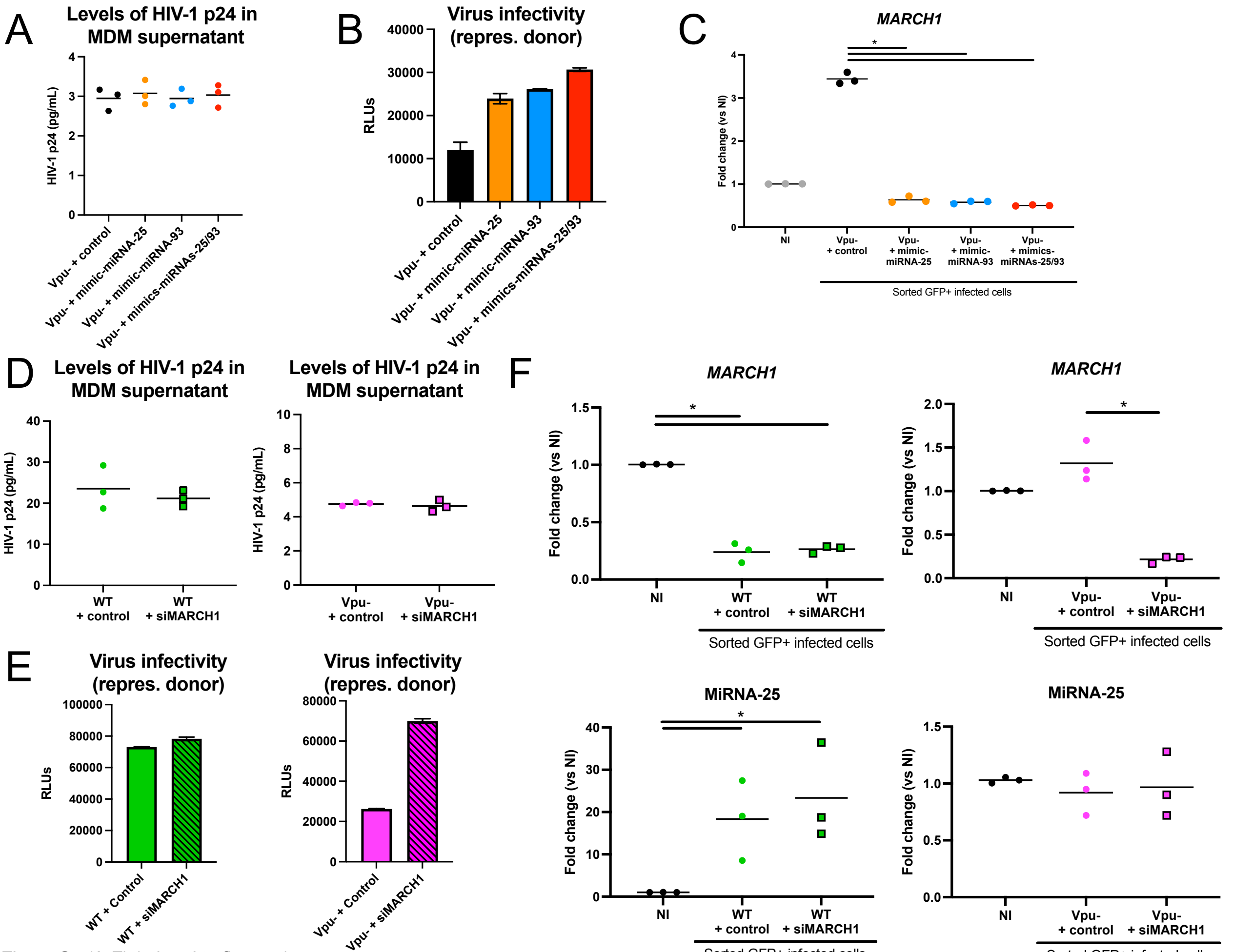

Figure S5 (A-F) (related to figure 7)

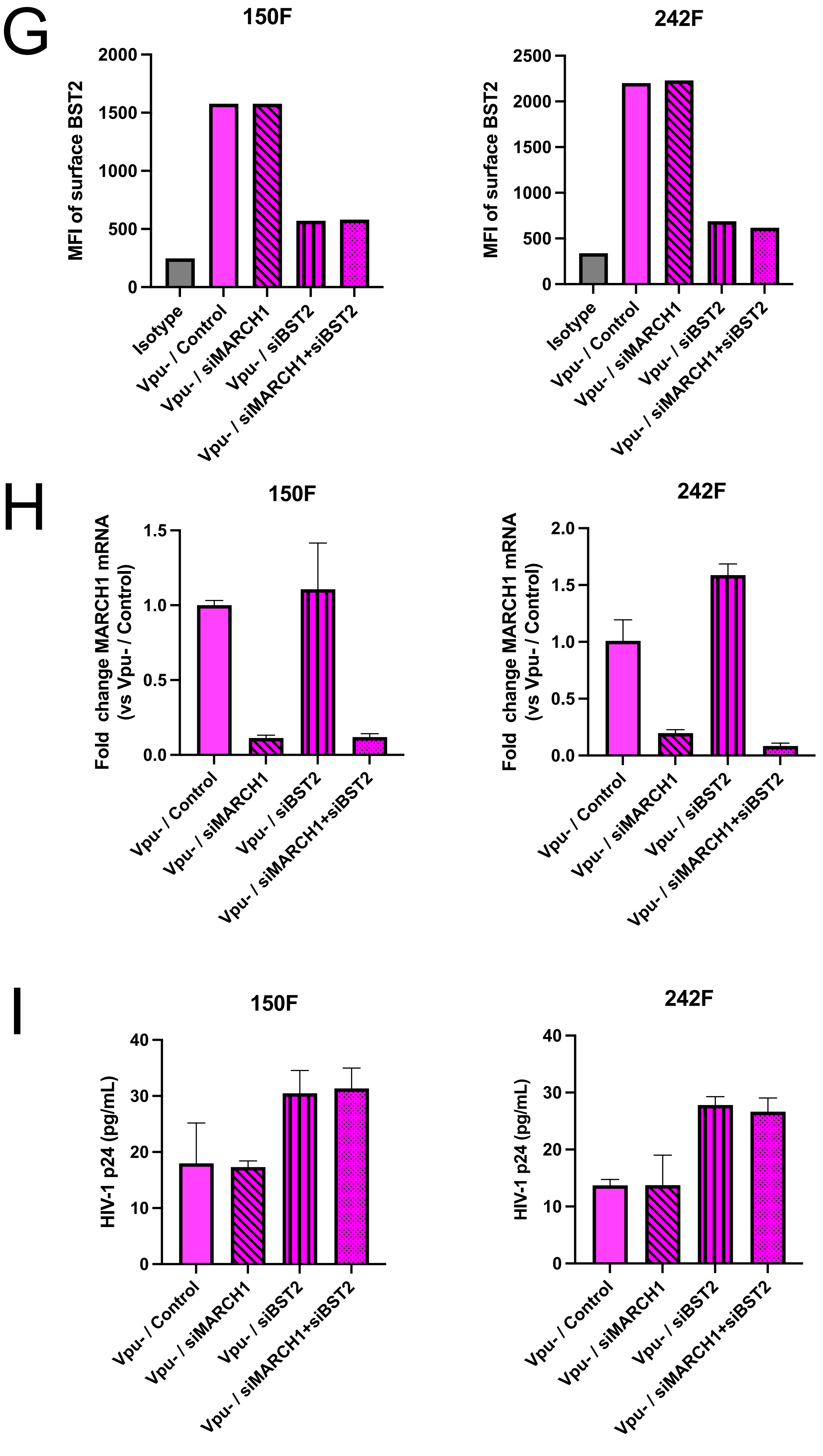

Figure S5 (G-I) (related to figure 7)

Figure S5; related to figure 7: A. Levels of HIV-1 p24 released from macrophages treated with control or the indicated mimics (related to figure 7A). B. Representative infectivity (1 ng of virus/normalized infection) data of viruses produced from MDMs of one blood donor shown in figure 7A. C. The level of *MARCH1* mRNA was determined by qRT-PCR in GFP-positive sorted infected MDMs previously treated with control or the indicated mimics (related to figure 7A). D. Levels of HIV-1 p24 released from macrophages treated with control or siRNAs for *MARCH1* and infected with either WT or Vpu-defective (Vpu-) HIV-1 viruses (related to figure 7B). E. Representative infectivity data from viruses produced from MDMs of one blood donor shown in figure 7B. F. Levels of *MARCH1* mRNA or miRNA-25 were determined by qRT-PCR in GFP-positive sorted infected macrophages previously treated with control or si*MARCH1* (related to figure 7B). G and H. Levels of cell surface BST2 protein (G) or *MARCH1* mRNA (H) in unsorted MDMs treated with either control or siRNAs for *MARCH1*, *BST2* or a combination of both and infected with Vpu-defective (Vpu-) HIV-1 viruses (related to figure 7C). I. Levels of HIV-1 p24 released from macrophages treated with either control or siRNAs for *MARCH1*, *BST2*, or a combination of both and infected with Vpu-defective (Vpu-) HIV-1 viruses (related to figure 7C). Bars represent mean±SD and *p= 0.05 using the Mann Whitney test.

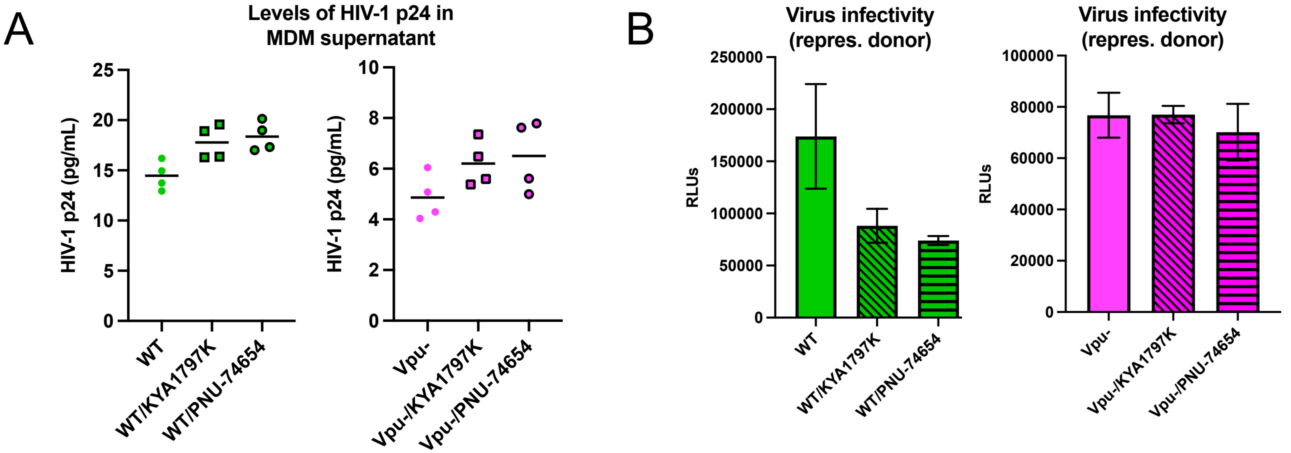

Figure S6 (related to figure 8)

Figure S6; related to figure 8: A. Levels of HIV-1 p24 released from macrophages treated with either the β-catenin inhibitors KYA1797K or PNU-74654 infected with Vpu+ or Vpu-defective (Vpu-) viruses. B. Representative infectivity data of Vpu+ or Vpu-defective (Vpu-) viruses produced from MDMs of one blood donor shown in figure 8D (normalized infection to 1 ng of virus; bars: mean±SD).
